# Identification and Functional Analysis of Temperate Siphoviridae Bacteriophages of *Acinetobacter baumannii*

**DOI:** 10.3390/v12060604

**Published:** 2020-05-31

**Authors:** Shimaa Badawy, Maria I. Pajunen, Johanna Haiko, Zakaria A. M. Baka, Mohamed I. Abou-Dobara, Ahmed K. A. El-Sayed, Mikael Skurnik

**Affiliations:** 1 Department of Bacteriology and Immunology, Medicum, Human Microbiome Research Program, Faculty of Medicine, University of Helsinki, 00014 UH Helsinki, Finland; shimaa_a_badwy@yahoo.com (S.B.); maria.pajunen@helsinki.fi (M.I.P.); 2 Department of Botany and Microbiology, Faculty of Science, Damietta University, 34511 New Damietta, Egypt; zakariabaka@du.edu.eg (Z.A.M.B.); aboudobara@du.edu.eg (M.I.A.-D.); akaelsayed@du.edu.eg (A.K.A.E.-S.); 3 Division of Clinical Microbiology, Helsinki University Hospital, HUSLAB, 00290 Helsinki, Finland; johanna.haiko@hus.fi

**Keywords:** *Acinetobacter baumannii*, temperate, bacteriophage, *Siphoviridae*, genome, *att* site, prophage, cohesive ends

## Abstract

*Acinetobacter baumannii* is an opportunistic pathogen that presents a serious clinical challenge due to its increasing resistance to all available antibiotics. Phage therapy has been introduced recently to treat antibiotic-incurable *A. baumannii* infections. In search for new *A. baumannii* specific bacteriophages, 20 clinical *A. baumannii* strains were used in two pools in an attempt to enrich phages from sewage. The enrichment resulted in induction of resident prophage(s) and three temperate bacteriophages, named vB_AbaS_fEg-Aba01, vB_AbaS_fLi-Aba02 and vB_AbaS_fLi-Aba03, all able to infect only one strain (#6597) of the 20 clinical strains, were isolated. Morphological characteristics obtained by transmission electron microscopy together with the genomic information revealed that the phages belong to the family *Siphoviridae*. The ca. 35 kb genomic sequences of the phages were >99% identical to each other. The linear ds DNA genomes of the phages contained 10 nt cohesive end termini, 52–54 predicted genes, an *attP* site and one tRNA gene each. A database search revealed an >99% identical prophage in the genome of *A. baumannii* strain AbPK1 (acc. no. CP024576.1). Over 99% identical prophages were also identified from two of the original 20 clinical strains (#5707 and #5920) and both were shown to be spontaneously inducible, thus very likely being the origins of the isolated phages. The phage vB_AbaS_fEg-Aba01 was also able to lysogenize the susceptible strain #6597 demonstrating that it was fully functional. The phages showed a very narrow host range infecting only two *A. baumannii* strains. In conclusion, we have isolated and characterized three novel temperate *Siphoviridae* phages that infect *A.* *baumannii*.

## 1. Introduction

*Acinetobacter baumannii* is a Gram-negative non-fermenting aerobic coccobacillus that plays a significant role in infecting patients admitted to hospitals [1,2]. *A. baumannii* is a relatively newly emerged pathogen, notorious for its role as prominent causative agents of nosocomial wound as well as community-acquired infections [3]. *A. baumannii* has become a challenge for modern medicine due to being one of the most important multiple-drug resistant (MDR) nosocomial pathogens. The antibiotic resistance mechanisms of *A. baumannii* include enzymatic inactivation of the drugs, modification of the drug target site, and active efflux or decreased influx of drugs [4]. Thus, the control and therapeutic management of *A. baumannii* has becomes a pressing concern [4,5,6]. Pan-resistant strains, resistant to all available antibiotics, have been encountered [7]. Carbapenems (imipenem and meropenem) and colistin (polymyxin B) antibiotics are the last resort for treatment of Gram-negative bacterial infections; but resistant strains have been widely reported in clinical settings [8,9]. Due to this threat, phage therapy has been considered, and already used, as an alternative treatment to manage infections caused by MDR pathogens [10,11], also recently in animals [12,13] and humans [14,15] against infections caused by MDR *A. baumannii*.

Bacteriophages (phages) are viruses that infect and reproduce in bacteria. The use of phages as therapeutic agents was initiated after their discovery by Frederick Twort in 1915 and Felix d’Herelle in 1917, where after, phages were used to treat a variety of bacterial infections all over the world [16,17]. Phages are highly host-specific [18], and their taxonomy is based on their morphology as well as on their nucleic acid [19,20]. Phages fall into two main classes.

- Virulent (lytic) phages follow the lytic infection cycle in which the phage overtakes the bacterial cell, reproduces itself at the expense of the host, and finally lyses and kills the host cell to release its progeny to the environment. This ability is the cornerstone of phage therapy [21].
- Temperate phages may enter the lysogenic cycle in which the phage genome integrates into the host genome using the activity of phage-encoded integrase, and the resulting bacteria are called lysogens. The integrated phage is called a prophage, it replicates as part of the host genome, and stays dormant for extended periods of time, due to the activity of a specific repressor that prevents the expression of the phage lytic cycle genes. The prophage may become induced and turn on the lytic cycle genes when the lysogen encounters adverse environmental conditions. The stress triggers the SOS (emergency) response, and the overexpression of proteases causes the degradation of the phage repressor thereby turning on the lytic cycle. The archetype of temperate phages is phage lambda [22].

Temperate phages which enter lysogeny maintain a long-term association with their host bacteria if they produce a mutually beneficial interaction that support efficient reproduction of both phages and bacteria [23]. The phages integration into the host genome is a crucial step in the lysogenic cycle and is mediated by the integrase protein, a DNA recombinase encoded by bacteriophages, at a distinct bacterial genome attachment site (*attB*), which is identical to an attachment site (*attP*) of the prophage genome [23]. Prophages do not integrate randomly into the host genomes. For example, prophages encoding a tyrosine integrase are usually integrated next to their host tyrosine tRNA gene and the palindromic structures of those temperate phages allow their integration within the bacterial genome [24].

Temperate phages are natural vectors for gene transmission among bacteria owing to their ability to integrate as prophages, and, consequently, affect the fitness and phenotype of the produced lysogen [25]. Several virulence factor or toxin encoding genes of pathogenic bacteria have been associated with prophages, indicating that temperate phages may play a significant role in increasing the host pathogenicity, on the other hand, a virulence-attenuating role is not often reported for temperate phages [26,27].

Whole genome sequences of several *A. baumannii* strains have revealed that they are polylysogenic, i.e., they harbor multiple integrated prophages [28]. Some prophages provide their host bacteria beneficial traits that increase the host fitness [29], some encode virulence factors including toxins that may benefit host pathogenesis [30] or antibiotic tolerance [31]. Prophages also protect the host bacteria against infection by other phages [32]. Resistance and other bacterial virulence elements are contained on highly mobile pieces of DNA that can easily spread to other bacteria. Prophages are one of the facilitators of this form of horizontal gene transfer, and have been discovered very often in bacterial genomes, offering advantageous features to the host. Prophages are claimed to be one of the major causes behind the evolution of *A. baumannii* pathogenicity [33]. Also remarkably, *A. baumannii* prophages encode for multiple putative virulence factors that may be implicated in the bacterium’s capacity to colonize host niches, prevent the host immune system, survive in unfavorable environments, and tolerate antibiotics. Overall the results point towards a significant contribution of prophages for the spreading and evolution of pathogenicity in *A. baumannii*, and highlight their clinical relevance [33].

Some phage therapy trials have failed in the past apparently due to lack of knowledge on phage biology and due to use of uncharacterized phages [34]. Therefore, there is still room for more studies to provide greater understanding of phage biology to avoid failures in phage therapy [35]. In our search for new *A. baumannii* specific bacteriophages we isolated three temperate bacteriophages with a very narrow host range belonging to the family *Siphoviridae*. The ca. 35 kb genomes were >99% identical to each other, contained 10 nt cohesive end termini, 52–54 predicted genes, an *attP* site and one tRNA gene each. We also present data that the phages were induced from two of the clinical strains used for the search and that one of the phages was able to lysogenize a susceptible strain demonstrating that it was fully functional.

## 2. Materials and Methods

### 2.1. Bacterial Strains, Media and Growth Conditions

The clinical *A. baumannii* and other bacterial strains used in this study (Table S1) originated from diverse patient samples (wound, tissue, sputum, broncho-alveolar lavage, trachea, pleural fluid, urine, bile, blood, rectum and drainage and intravenous catheters) isolated at Department of Bacteriology, University of Helsinki and Helsinki University Central Hospital (HUSLAB), Helsinki, Finland. The *A. baumannii* strain DSM 106838 (storage number #6597, Table S1) was received from DSMZ, the German Collection of Microorganisms and Cell Cultures GmbH. The bacteria were grown with shaking at 220 rpm in lysogeny broth (LB) [36] medium at 37 °C overnight (16–24 h). Luria agar (LA) plates contained LB supplemented with 1.5% agar and soft agar contained LB supplemented 0.4% agar. All strains were stored in tryptic soy broth containing 20% glycerol at −80 °C. The strains are identified in the text by their storage numbers (Table S1).

### 2.2. Bacteriophage Isolation and Propagation

Enrichments of *Acinetobacter* specific bacteriophages were carried out as described [37] with some modifications. Twenty clinical *A. baumannii* strains were grown separately at 37 °C for 16 h and 100 µL of each culture was combined into two sets of parallel pools with 10 strains each. One mL of a sterile-filtered (0.45 µm) sewage filtrates was mixed with each pair of pools to which 9 mL of LB was added. These pooled enrichment cultures were incubated overnight on a rocking platform at 37 °C to allow enrichment of any *Acinetobacter*-specific phages present. To each 3 mL portion of the enrichment cultures 0.2 mL of chloroform was added to kill and lyse the bacteria, and the mixture was shaken for 20 min at room temperature (RT). The chloroform-treated lysates were then clarified by centrifugation at 5000 rpm for 10 min at 4 °C, and the supernatants filtered through 0.45 µm filters (Minisart^®^ Sartorius, Göttingen, Germany) to remove bacterial cells. The obtained lysates were then stored at 4 °C for bacteriophage isolation.

### 2.3. Bacteriophage Titration by Droplet and Double-Agar Overlay Methods

One hundred µL of indicator bacteria (OD_600_~1.0) was mixed into 3 mL of molten soft agar (adjusted to 50 °C) and immediately poured on warm LA plates. The soft agar was allowed to solidify at RT for 20 min. The enrichment lysates or phage suspensions to be tested were serially 10-fold diluted in SM buffer (50 mM Tris-HCL, 100 mM NaCl, 8 mM MgSO_4_·7H_2_O, 0.01% gelatin, pH 7.5). Five µL droplets of the undiluted and diluted suspensions were pipetted on the solidified soft agar plates that were incubated overnight at 37 °C. The plates were observed for the presence of bacterial lysis under the droplets to indicate the presence of phages [38].

For double-agar overlay titration method appropriate dilutions of the phage suspension, estimated based on the droplet titration results, were prepared to contain 50–500 plaque forming units (PFU) per 50 µL. The 50 µL phage aliquots and 100 µL of indicator bacteria (OD_600_~1.0) were pipetted into 3 mL of 50 °C-adjusted soft agar, carefully mixed and poured on LA plates. After overnight incubation the individual plaques were enumerated and the original phage titers (PFU/mL), calculated based on the plaque numbers and the dilution factors [18,39].

### 2.4. Plaque Purification

Individual plaques on double-agar overlay plates were used for plaque purification of individual phages. A well-isolated single plaque was punched out using a sterile Pasteur pipette and transferred to an Eppendorf tube containing 500 µL of SM buffer and 50 µL of chloroform. The tube was shaken for 20 min at RT, centrifuged at 11,000× *g* for 10 min at 4 °C, and 300 µL of the water phase was transferred to a new Eppendorf tube that was kept cap open in a laminar-flow safety cabinet to evaporate the residual chloroform. The phage concentration was titrated, and single plaque purification was repeated from new double agar overlay plates at least five times to ensure purity of each phage [40].

### 2.5. Preparation of Phage Stocks

Phage stocks were prepared from semiconfluent soft agar plates prepared using appropriately diluted purified phage solutions. Plates were incubated overnight, and 3 mL of SM buffer was added on the semiconfluent lawn of plaques and incubated at RT for 30 min on a rocking platform. The soft agar and the buffer were collected into a 15-mL polypropylene (PP) tube, and the remaining soft agar was recovered by repeating the procedure. Two hundred µL chloroform was added to every 3 mL of the combined soft agar samples, the tubes were mixed for 15–20 min at RT, and centrifuged at 5000× *g* for 10 min at 4 °C. The supernatant was filtered through a 0.45-µm filter (Minisart^®^ Sartorius) into a sterile 15-mL PP tube, and 40% sucrose was added to final concentration of 8%. The obtained stocks were titrated and stored at 4 °C [41,42].

### 2.6. Phage Purification from Liquid Lysates

Large scale lysates of phages were prepared in 500 mL cultures. *A. baumannii* strain #6597 (Table S1) served as a host strain for the propagation of phages. Phages were precipitated from the lysate according to [43] with some modifications. DNase I and RNase were added, each to final concentration of 1 μg mL^−1^, to the lysate that was incubated for 30 min at RT. Solid NaCl (29.2 g) was dissolved into the lysate (1 M final concentration) that was incubated on ice for 1 h prior centrifugation at 11,000× *g* for 10 min at 4 °C to remove bacterial debris. PEG-8000 was slowly stirred into the supernatant to a final concentration of 10% at RT after which the solution was incubated at least 1 h at 4 °C to precipitate the phage particles. The precipitated phages were pelleted by centrifugation at 11,000× *g* for 10 minutes at 4 °C. After carefully removing all liquid from the centrifuge bottles, the phage pellet was resuspended gently into 8 mL SM buffer. To remove residual PEG, the suspension was extracted thrice with an equal volume of chloroform followed by centrifugation at 3000× *g* for 15 min at 4 °C. The phage preparations were further purified by discontinuous glycerol density gradient ultracentrifugation through 5% and 40% glycerol layers in TM buffer using the Beckman BSW55Ti rotor at 40,000 rpm at 4 °C for 3 h [44]. The phage pellet was resuspended in SM buffer containing 8% sucrose.

### 2.7. Transmission Electron Microscopy

A 3 µL drop of purified high-titer (10^10^–10^13^ PFU/mL) phage in 0.1 M ammonium acetate, pH 7.0, concentrated using a 10^5^ Da cut-off Vivaspin ultrafiltration (Minisart^®^ Sartorius), was deposited on carbon-coated 400 mesh copper grid and allowed to adsorb for 1 min, followed by 2% uranyl acetate for 30 s [45]. Samples were examined with a JEOL JEM-1400 transmission electron microscope JEOL Ltd., Tokyo, Japan) under 80 kV at the electron microscopy unit (Institute of Biotechnology, University of Helsinki, Helsinki, Finland). Pictures were taken using Gatan Orius SC 1000B bottom-mounted charged coupled device (CCD)-camera (Gatan Inc., Pleasanton, CA, USA). The dimensions of at least 10 phage particles measured to calculate the average and standard error values.

### 2.8. Phage Adsorption Assay

Adsorption is the first step of phage infection of host bacteria. Approximately 3 × 10^5^ PFU in 100 µL was mixed with a 500 µL of *A. baumannii* strain #6597 grown to OD_600_ ~ 1–1.2 in LB, and to a control tube with 500 µL sterile LB. The tubes were incubated at 37 °C and at different time points (1, 5, 10, 15, 20, 25, and 30 min) the tubes were centrifuged at 16,000× *g* for 3 min and the supernatants placed immediately on ice until titrated for non-adsorbed phages. The phage titers were compared to that of the control supernatant set as 100%. Each assay was performed in duplicate and repeated for 3 times.

### 2.9. Phage Host Range Determination

The host specificity of the phages was determined by the droplet titration method (described above) on 91 strains (Table S1) including 21 *A. baumannii*, 18 *A. pittii,* one *A. calcoaceticus,* one *A. junii*, three *A. lwoffii*, three *A. nosocomialis,* two *A. radioresistens*, three *A. ursingii*, one *Acinetobacter sp*, 24 *Escherichia coli*, 10 *Staphylococcus aureus*, two *Pseudomonas aeruginosa,* one *Yersinia pseudotuberculosis* and one *Klebsiella pneumoniae* strain(s). Positive droplet test results were confirmed by the double-layer method using appropriately diluted phage preparations.

### 2.10. Phage and Bacterial DNA Extraction

Phage DNA isolation was performed from 10^10^–10^13^ PFU/mL phage preparations using the phenol-chloroform extraction method [43] with some modifications. Briefly, to 400 µL of the phage suspension 1.3 µL DNase I (1 U/µL, Promega, Madison, WI, USA) and 4 µL RNase A (1 mg/mL) were added and the mix was incubated 30 min at 37 °C to degrade bacterial DNA and RNA. Then, 16 µL of 0.5 M EDTA, 1.2 µL of proteinase K (20 mg/mL), and 20 µL 10% SDS were added to the tube that was incubated at 56 °C for at least 1 h. The cooled suspension was extracted sequentially with a volume of phenol, phenol/chloroform (1/1) and chloroform, each time mixing the tube gently for 15 min, followed by 5 min centrifugation at 16,000× *g*. The nucleic acid was precipitated from the final aqueous phase by adding 0.1 volumes of 3 M sodium acetate pH 5.2, and 2 volumes of absolute ethanol. The tube was mixed manually for 2–3 min until the precipitated DNA thread became visible, and could be transferred using a 1 µL inoculation loop into a tube containing 1 mL 70% EtOH. After 10 min centrifugation at RT, the supernatant was carefully removed and the pellet was air dried and dissolved overnight into 50–100 µL of TE buffer (10 mM Tris-HCL, 1 mM EDTA, pH 8.0). In the case that the DNA thread failed to form, the DNA was pelleted by centrifugation, and washed with 1 mL of 70% EtOH as above. The isolation and purification of the genomic DNA from bacterial strains was accomplished using JetFlex Genomic DNA Purification Kit (Thermo Fisher Scientific, Waltham, MA, USA) following the manufacturer’s instructions. The quality and quantity of the DNA was estimated using the NanoDrop spectrophotometer (ND-1000, Wilmington, DE, USA) and/or the Qubit machine (Invitrogen Qubit 2.0 Fluorometer, CA, USA) applying the QubitTM dsDNA BR Assay Kit (Thermo Fisher Scientific), followed by visualization by agarose gel electrophoresis [40].

### 2.11. Genome Sequencing, Assembly and Bioinformatics Based on Sequence Analysis

The genomic DNA of the phages and of the bacterial strains was sequenced at Eurofins Genomics (https://www.eurofinsgenomics.eu/) using Illumina HiSeq with 150-bp paired-end reads. The DNA was fragmented using sonication into 300–600 bp fragments that were then processed to generate DNA libraries. The obtained sequence reads were assembled de novo using the A5-miseq pipeline [46]. The read coverage of the resulting contigs were checked with the Artemis software [47,48] and the contaminating bacterial genomic sequences identified by their >100-fold lower read coverages (the read coverage of the phage contig was >10,000) and BLASTN searches. To verify the fidelity of the assemblies, the reads were mapped back to the de novo assembled contigs using the Geneious (Biomatters Ltd., Auckland, New Zealand) prime R10 software version 2019.0.4. tools [49]. Preliminary annotation of the phage genomes were carried out using rapid annotation subsystems technology (RAST) [50] that was manually checked and revised with the Artemis software [47,48]. The PhageTerm program was used to identify the termini of the phage genomes [51]. The identities and functions of the predicted genes and gene products were analyzed using the BLASTP (https://blast.ncbi.nlm.nih.gov/Blast) and HHpred [52] servers. Different EMBOSS sequence analysis tools were used through the Chipster platform [53] at the Centers for Scientific Computing (https://www.csc.fi/). The multiple sequence alignment program MAFFT was used to compare phage genome sequences [54]. The phylogentic trees were constructed using the Molecular Evolutionary Genetic Analysis Software, Version X (MEGA X), where the neighbor-joining statistical tree analysis method was used. The number of bootstrap replications was set at 1000. The Poisson substitution model was used within the MEGA X software [55]. The phage and prophage whole genome sequence phylogenetic tree was constructed using the VIrus Classiﬁcation and Tree building Online Resource (VICTOR) [56]. The *A. baumannii* strain multilocus sequence types (MLST) were determined using the PubMLST web service (https://pubmlst.org/abaumannii/) and the O- and K-serotypes using the Kaptive service [57].

### 2.12. PCR and Sanger Sequencing

The PCR primers (Table S2) were designed using the EMBOSS Eprimer3 tool in Chipster [53], and commercially synthetized at Metabion International AG (Steinkirchen, Germany). The PCRs were performed in 0.2 mL thin walled PCR tubes (*4titude*^®^ Ltd, Wotton, UK), in a total volume of 25 μL containing 1 μL of DNA template, 0.2 μM of each primer (Table S2), 200 μM of dNTP mix (Thermo Fisher Scientific), 2.5 μL of 10× Standard Taq Reaction Buffer and 1.25 U of Taq DNA Polymerase (Thermo Fisher Scientific). The PCR cycling included an initial denaturation at 95 °C for 3 min, followed by 34 cycles each consisting of denaturation at 95 °C for 30 s, 30 s at annealing temperature, and extension for 30 s at 72 °C. This was followed by a final extension step at 72 °C for 5 min after which PCR products were kept on hold, at 4 °C, until further processing. The annealing temperatures were calculated using the net service at https://www.thermofisher.com/. The PCR products were analyzed using 1% agarose gel electrophoresis and cleaned using the Nucleospin Gel extraction and PCR clean-up kit (Macherey-Nagel GmbH, Düren, Germany). The purified PCR fragments were sequenced with appropriate sequencing primers using the Sanger sequencing service at the Institute for Molecular Medicine Finland (https://www.fimm.fi/en/services/technology-centre/sequencing).

### 2.13. Restriction Endonuclease Analysis

Restriction digestions were carried out using the restriction enzymes *Eco*RI, *Hinc*II, *Nsi*I, *Pst*I, *Sal*I, *Sca*I, *Sex*AI, and *Spe*I (Thermo Fischer Scientific). These enzymes gave several well separated bands when in silico digested by the NEBcutter (http://nc2.neb.com/NEBcutter2/). The digestions were carried out in a final volume of 10 µL, containing DNA (ca. 300 ng), 0.5 µL of enzyme and 1 µL of Fast digest green buffer (10×, Thermo Fisher Scientific). After 2–16 h incubation at 37 °C the restriction fragments along with undigested DNA and GeneRuler 1 kb DNA Ladder (Thermo Fisher Scientific) were loaded on 1% (*w*/*v*) agarose gel including 0.005% (*w*/*v*) Midori green. After the electrophoresis the fragments were visualized using UV transillumination and images recorded using the BioRad GelDoc XR+ imaging system.

### 2.14. Lysogenization Experiment

To test the ability of the phages to form lysogens, phage resistant colonies were isolated and prophage inductions were performed as described [58] with some modifications. The prophage-free *A. baumannii* strain #6597 was used as a host for the lysogenization test [59]. Bacterial suspension was spread on LA plate, allowed to dry for a while, and 10 µL drops of phage stock were pipetted on the bacterial lawn. After 48 h incubation at 37 °C, ten surviving colonies were selected randomly from within the lysis zones, further tested for phage resistance. To confirm the integration of the phage genome, bacterial DNA isolated from the confirmed phage-resistant strains was used as template in phage-specific PCRs as described above.

### 2.15. Prophage Induction Experiments

Spontaneous prophage induction from *A. baumannii* strains #5707 and #5920 was carried out by inoculating a single colony from each strain separately in 5 mL of LB, incubating overnight at 37 °C. The culture was split into two and to one portion of the cultures, 0.2 mL of chloroform was added to kill and lyse the bacteria, and the mixture was shaken for 20 min at RT. The chloroform-treated lysates and non-treated cultures were then clarified by centrifugation at 5000 rpm for 10 min at 4 °C, and the supernatants filtered through 0.45 µm filters (Minisart^®^ Sartorius) to remove bacterial cells. The obtained samples were then stored at 4 °C for further experiments.

### 2.16. Nucleotide Sequence Accession Numbers

The annotated nucleotide sequences of the phage genomes were submitted to sequence databases under the following accession numbers: fEg-Aba01 (MT344103), fLi-Aba02 (MT344104) and fLi-Aba03 (MT344105). The raw sequence read data of *A. baumannii* strains #5707, #5907, #5920 and #6597 was deposited to NCBI sequence read archive under bioproject PRJNA625727, and received the accession numbers SRX8124491, SRX8124492, SRX8124492, and SRX8124494, respectively.

## 3. Results

### 3.1. Phage Isolation and Phenotypic Characterization

Enrichments were carried out in pools of ten clinical *A. baumannii* strains each and the obtained sterile-filtered pool-lysates were then tested for phages using each clinical *A. baumannii* strain individually as indicator bacteria in the soft agar plates. Phage activity was detected only against one of the strains, the *A. baumannii* strain #6597 (Table S1). After several rounds of plaque purification from different lysates, three phages named vB_AbaS_fEg-Aba01, vB_AbaS_fLi-Aba02 and vB_AbaS_fLi-Aba03 (hereafter referred to as fEg-Aba01, fLi-Aba02 and fLi-Aba03, respectively) were obtained.

**Figure 1:**
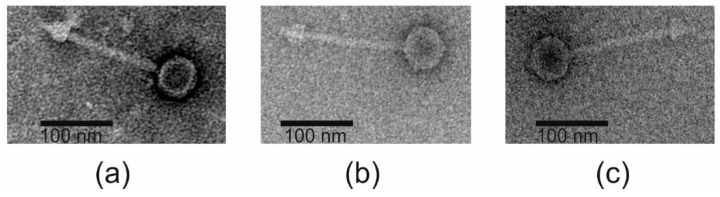
Transmission electron micrographs of fEg-Aba01 (**a**), fLi-Aba02 (**b**), and fLi-Aba03 (**c**). The phages were negatively stained with 2% uranyl-acetate. The scale bar is 100 nm.

The three phages showed very similar plaque morphologies and formed round slightly turbid 1–1.5 mm diameter plaques with haloes on *A. baumannii* #6597 (Figure S1). Transmission electron microscopy revealed that all the phages resemble morphologically *Siphoviridae* (Figure 1). The physical dimensions of the phages are listed in Table 1. The phages possess icosahedral capsids (58–63 nm in diameter) to which a long, flexible and non-contractile tail (136–156 nm in length and 8–9 nm in diameter) was attached (Table 1).

**Figure 2:**
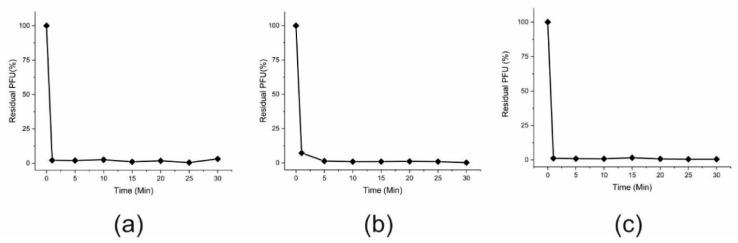
Adsorption curves of fEg-Aba01 (**a**), fLi-Aba02 (**b**), and fLi-Aba03 (**c**) to *A. baumannii* #6597. The phage titer in the control supernatant was set to 100% and the residual titers at different time points were related to that. Each assay was performed in duplicate and repeated for 3 times. Note that the standard deviation bars are mostly hidden behind the symbols.

Adsorption is the first decisive step of phage infection of host bacteria. The adsorption curves of the phages showed that the phages fEg-Aba01, fLi-Aba02 and fLi-Aba03 adsorbed extremely rapidly onto *A. baumannii* #6597 bacteria. Within 1 min >98% of the free phage particles had already adsorbed to the host bacteria, and at 5 min nearly 100% were adsorbed (Figure 2).

The host ranges of phages fEg-Abo01, fLi-Aba02 and fLi-Aba03 were tested with 91 strains representing 14 species (Table S1). The phages infected only the original *A. baumannii* host strain #6597, and in addition, a recent MDR *A. baumannii* patient isolate, strain #6898 (Table S1).

### 3.2. Phage Genomic Characterization, Annotation and Comparison to Other Phages

Each de novo assembly of the Illumina sequencing reads of the phages resulted in >100 contigs. While for each phage, one contig, 33–35 kb in size, had very high read coverage, the read coverage of the other contigs was low. In addition, as the BLASTN searches with the low-coverage contigs revealed that they represented *A. baumannii* genomic sequences, it is very likely that they represent host bacterial DNA sequences either as contaminants in the phage DNA samples or through transduction. Thus the high-coverage contigs represented the phage genomic sequences, and the low-coverage contigs were excluded from further study.

**Figure 3:**
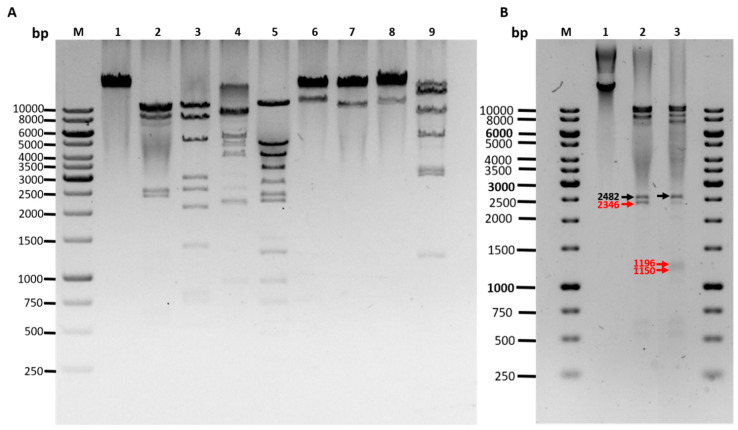
Agarose-gel-electrophoresis analysis of restriction enzyme digested fEgAba01 DNA. Panel (**A**) fEg-Aba01 genomic DNA digested with *Eco*RI (lane 2), *Hin*cII (lane 3), *Nsi*I (lane 4), *Pst*I (lane 5), *SaI*I (lane 6), *Sca*I (lane 7), *Sex*AI (lane 8), and *Spe*I (lane 9). Lane 1, undigested DNA. Lane M, 1-Kb DNA ladder. Panel (**B**), confirmation of the cohesive ends. Lanes: 1, undigested phage DNA; lane 2, *Eco*RI-digested DNA, the red arrow indicates the joint fragment; lane 3, *Eco*RI-digested phage DNA, heated after digestion at 80 °C for 15 min, followed by rapid cooling to room temperature (RT). The red arrows indicate the left and right end *Eco*RI fragments. The black arrows indicate the 2482 bp fragment.

The three phage sequences were highly similar (>99% identical) with each other, however, they showed no similarity to any phage genomes deposited in the sequence databases. The restriction digestion patterns of the three phages were almost identical (two shown in Figure S3). Comparison of the sequence-based and experimental restriction digestion patterns allowed rough mapping of the physical ends of the phage genomes, however, the PhageTerm analysis allowed exact identification of the ends, and it revealed that the three phages each contained 10 nt long 3’-cohesive ends with identical sequences: 5’-CGCCCCCCAT-3’ (Figure S4). To confirm the presence of the cohesive ends, *Eco*RI digested phage DNA was analyzed in agarose gel after heat treatment at 80 °C and either cooled rapidly on ice or allowed to cool slowly to RT. The cohesive ends in the latter case annealed to each other and produced a visible larger band in the gel (Figure 3B). Based on these results the genome sequences were rearranged to represent the linear genomes in the phage particles and to locate the majority of the predicted genes to the forward direction. Finally, the experimental restriction enzyme digestions of the phage DNA were in perfect match with in silico restriction digestion fragment sizes (Figure 3A) that are as follows:

- *Eco*RI (10231, 8997, 8427, 2482, 1196 left end, 1150 right end, 604, 522, 170 bp)
- *Hin*cII (9559, 8170, 5179, 2713, 2546, 2074, 1228 right end, 767, 711, 496, 219, 117 left end)
- *Nsi*I (9006, 8804, 6595, 3916, 2482 right end, 2104, left end, 872)
- *Pst*I (11219, 5337, 4003, 3313, 2405, 2240, 1474 left end, 1295 right end, 912, 699, 444, 438)
- *SaI*I (22168 right end, 11611 left end)
- *Sca*I (23619 right end, 10160 left end)
- *Sex*AI (22976 right end, 10803 left end)
- *Spe*I (13517 left end, 7659, 5298, 2926, 2907, 1185, 287 right end)

**Figure 4:**
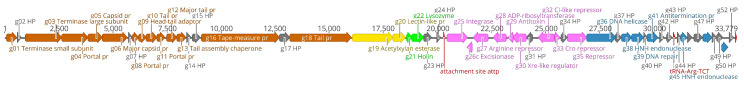
The annotated genome map of fEgAb01. The predicted genes are shown as colored arrows labelled with predicted functions (Genes encoding structural proteins, brown; lysogen functions, purple; nucleic acid enzymes, blue; hypothetical proteins, grey; tRNA gene and the 3’-cohesive end, red; lysis functions, green; carbohydrate interactions, yellow; for details see Table S3). The map was drawn with Geneious 10.2.6 (www.geneious.com). HP, hypothetical protein; pr, protein.

Annotation of the sequences showed that fEg-Aba01 consisted of 52, fLi-Aba02, of 53, and fLi-Aba03, of 54, predicted genes (Table 1 and Table S3). Seven of the genes in each were encoded in the reverse strand. The GC contents of the phages were similar, 40.1–40.2% (Table 1) that is very close to the GC content of *A. baumannii* host, ca. 39% [60]. The overall organization of the genome of fEg-Aba01 is presented in Figure 4.

While no similar phages were found from nucleotide sequence databases, a 99% identical 35 kb genomic island (GI5) annotated as a prophage is present in the *A. baumannii* strain AbPK1 genome (GenBank accession number CP024576.1). The AbPK1 strain was isolated as the causative agent of virulent epidemic pneumonia that killed hundreds of sheep on a farm in Pakistan [60]. In addition, the predicted CP14 prophage of three other *A. baumannii* strains (AB042, AB043 and ATCC17978, acc. no CP019034, CP043910, CP018664, respectively) showed 80% identity to fEg-Aba01 [61].

**Figure 5:**
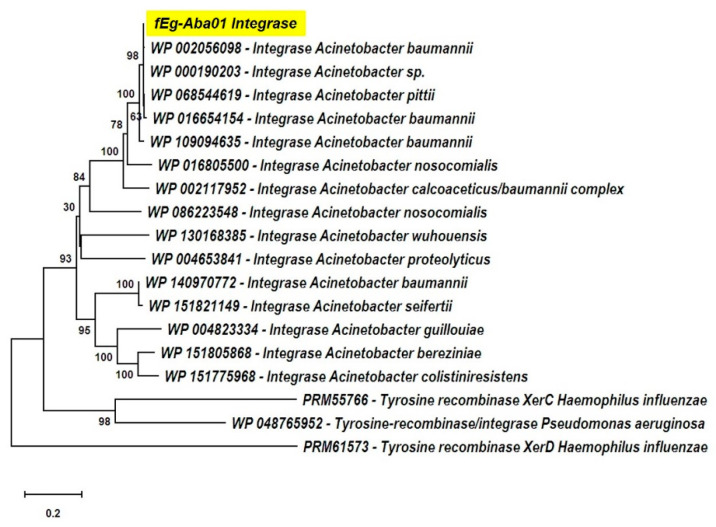
The phylogenetic tree of fEgAb01 integrase with different site-specific Acinetobacter integrases and rooted with the *Haemophilus influenza* and *Pseudomonas aeruginosa* integrases. The tree was constructed using the MEGA X neighbor-joining statistical tree analysis method with bootstrapping set to 1000 tree branches are proportional to branch lengths. The branch labels represent bootstrap values. The integrase of fEg.Aba01 is highlighted in yellow.

The predicted functions of the phage gene products, based on database searches, are shown in Table S3. A putative function could be assigned to ca. 30 gene products leaving 22 annotated as hypothetical proteins. Five functional groups were identified:

1. The lysogen decision gene clusters of the three phages were identified and the gene products annotated based on similarity searches (Table 2). The integrase (Gp25 in fEg-Aba01; Gp27 in fLi-Aba02 and fLi-Aba03) is 100% identical in all three phages. It is the site-specific recombinase that by catalyzing recombination between the two DNA molecules results in either integration or excision of the prophage into and from the host chromosome [62]. The arginine (Gp27c; Gp29c) and the CI-like (Gp32c; Gp34c) repressors account for the establishment of lysogeny and the immunity of lysogens to superinfection. The production of CI represses the Cro (Gp33; Gp35) synthesis. The excisionase (Gp26c; Gp28c) binds to the integrase and enables it to reverse the integration process and liberate the prophage. The Xre family transcriptional regulator (Gp30c; Gp32c) and another repressor (Gp35; Gp37) may also be involved in the regulation of lysogeny/lysis decision. Traditionally, the lysogeny/lysis decision is regulated by the competition between CI repressor and Cro proteins for the occupancy of the operator (OR) region. The Cro protein binds to the OR and OL promotors and turns off the transcription of the CI repressor gene and thus allows transcription and translation of the lytic cycle and other late genes [63]. Some integrases are associated in the integration and excision of phage genomes, whilst others are essential for the maintenance of plasmid copy number or elimination of chromosomal dimers [64,65,66]. A phylogenetic tree of *Acinetobacter* site-specific integrase sequences was constructed (Figure 5). The results demonstrate that Gp25 integrase clusters among the bacteriophage Hp1-like tyrosine integrases in the XerC superfamily. The ADP-ribosyltransferase (Gp28c; Gp30c) and the antitoxin (Gp29c; Gp31c) may be a toxin-antitoxin pair stabilizing the lysogen [67]. The hypothetical protein (Gp31c; Gp33c) is similar to a *A. baumannii* protein that shares a fold with a cell division regulator protein of *Streptococcus pneumonia* GbsP [68] and may interact with the bacterial cell wall synthesis.
2. The DNA synthesis, regulation and replication gene products include the Xre family transcriptional regulator (Gp30c; Gp32c), the DNA helicase (Gp36; Gp38) that unwinds the DNA to create template for DNA replication [69], and another repressor (Gp35; Gp37) likely involved in gene regulation. The putative HNH endonucleases (Gp38, Gp45 and Gp51; Gp40, Gp47 and Gp53) may play a variety of roles in replication, recombination, and repair pathways [70]. Finally, the antiterminator protein (Gp41; Gp43) renders RNA polymerase resistant to termination signals [71].
3. The DNA packaging into the phage head is carried out by the terminase complex containing the small and large terminase subunits (Gp01 and Gp03, respectively). It recognizes the *cos* site, introduces there the nicks to generate the cohesive ends to the genome and separates the cohesive ends in a reaction requiring ATP hydrolysis [72]. The terminase and the phage portal proteins (Gp04, Gp08 and Gp11) are believed to be the initiators of the head assembly. The NinB homolog (Gp48; Gp50) may be associated in recombination.
4. The phage structural proteins are encoded by the genes encoded in the left half of the genome (Figure 4), and include Gp05 and Gp06 that are annotated as capsid and major capsid proteins, respectively, that initiate formation of the procapsid [73], Gp09 is the putative phage head-tail adaptor, Gp16, the tail length tape-measure protein, and Gp12, the major tail protein. Gp8 and Gp10 are also structural peorteins, and Gp18 (Gp20 in fLi-Aba02 and fLi-Aba03) is also predicted to be a tail component (Table S3). Gp13 is a putative tail assembly chaperone. The predicted tail length based on the tape measure protein of 1195 amino acid residues, 1.5 Å per residue [74], would be ca 180 nm, that is 20–30 nm longer than the measured tail lengths (Table 1). Perhaps the tape measure protein extends to the tip of the bulky tail baseplate omitted from the measures.
5. The host lysis protein holin is predicted to be Gp21 (Gp23 in fLi-Aba02 and fLi-Aba03), and Gp22 (Gp24 in fLi-Aba02 and fLi-Aba03), the lysozyme, involved in the bacterial lysis and release of the phage progeny [75]. The predicted peptidoglycan hydrolase (Gp19 in fLi-Aba02 and fLi-Aba03) encoding gene is not present in fEg-Aba01, actually, it is deleted and the *g18* in fEg-Aba01 is a fusion product containing 5’-end of gene *g18* and 3’-end of gene *g20* of fLi-Aba02 (Table S3). The acetylxylan esterase and lectin-like proteins (Gp19;Gp21 and Gp20;Gp21, respectively) are both carbohydrate-associated proteins and might have a function in degrading the bacterial capsule polysaccharides.

Multiple genome alignment of the three phages and the prophage sequences of the *A. baumannii* strain AbPK1 (from CP024576.1) and those of strains #5707 and #5920 revealed several regions of differences (RoD) with overview in Table 3 and detailed illustration in Figure S5. The alignment demonstrates that all the genomes resemble closely each other (Figure S5A) and that local deletions/insertions/substitutions have taken place (Figure S5B). The RoD-02 deletion of 832 bp in fEg-Aba01 deleted the predicted peptidoglycan hydrolase gene (*g19* in fLi-Aba02) and generated the fusion gene *g18* in fEg-Aba01. Visual inspection of the RoDs (Figure S5B) also demonstrated that single nucleotide substitutions were mainly present in the AbPK1 prophage sequence (RoD-03, RoD-06 and RoD-08) suggesting that it is more distantly related to the others, this is also evident from the whole-genome phylogenetic tree (Figure S5A) where fEg-Aba01 is an outgroup and the other five sequences cluster together. Remarkably, the fLi-Aba02 was almost identical with the prophages of strains #5707 and #5920 while fLi-Aba03 differed from those only in RoD-06. The fEg-Aba01 genome differed from the others based on the deletions in RoD-01, RoD-02, RoD-05 and RoD-05 that all caused severe rearrangements.

### 3.3. Prophage Lysogenic Characteristics

This suggested that the origin of the phages could be a prophage in any of the twenty clinical *A. baumannii* strains used in the enrichment experiment. To this end we designed two phage-specific primer pairs to detect the presence of the putative prophages by PCR and used the isolated DNA from each of the twenty clinical *A. baumannii* strains as template. Phage genome DNA was used as a positive control. Three strains (#5707, #5907 and #5920) gave strong positive PCR results with both primer pairs (Table S4).

To confirm these results the genomic DNA of these strains together with strain #6597 were sequenced and the obtained raw read data was used in de novo assembly, and the reads were also mapped directly to the three phage genomes used as references. These results confirmed that the strains #5707 and #5920, but not #5907, carried the whole prophage (Figure S6). Based on the whole genome sequence data the MLST, capsular (K) and lipo-oligosaccharide (LOS) types were determined for the strains (Table 4). These data shows that the prophage-positive strains #5707 and #5920 are very similar as they both have the same MLST, and LOS and capsule types. The other two strains differ from the two and from each other. BLASTN alignment of the sequence data of the strains against the prophage locus of AbPK1 (Figure S5) shows that strain #5907 carries a truncated prophage genome while the prophage sequences are completely absent from strain #6597. The truncated prophage corresponds to AbPK1 nucleotides from 2546100 to 2560500 (acc. no. CP024576.1), explaining the positive 700 bp PCR result for this strain (Table S4). Interestingly though, the location of the truncated prophage sequences of #5907 were not the same where the prophage is located in AbPK1.

In a population of lysogenic bacteria a few at times enter the lytic cycle and phages are released in small quantities into the growth medium. To detect this experimentally the clinical strains #5707 and #5920 were grown in liquid medium to stationary phase. The presence of strain #6597 specific phages was detected from the culture supernatants of both lysogenic strains confirming that the strains very likely were the origin of the phages (data not shown).

**Figure 6:**
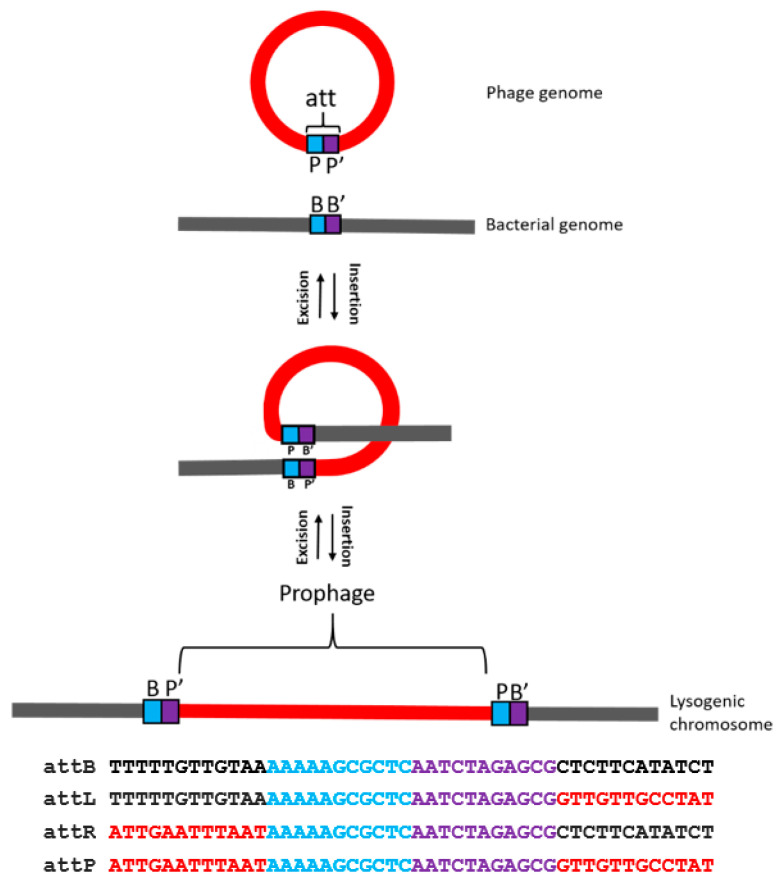
The prophage (red), flanked by *attL* and *attR* upon insertion into *A. baumannii* genome (black). The attachment site in blue and violet [**AAAAAGCGCTCAATCTAGAGCG**] is the cross-over site for phage integration and excision.

The presence of the prophage in the genomic sequences of the three *A. baumannii* strains, i.e., of AbPK1 (from CP024576.1) and of strains #5707 and #5920 (this work) allowed the identification of the attachment site (*attB*) in the bacterial genome as it is duplicated upon the integration of the phage genome. Thus, the identical attachment site sequences are present in both the phage and the host genomes. The phage-encoded integrase enzyme, with the aid of a special host protein, catalyzes site-specific recombination, i.e., the physical exchange of viral and bacterial DNA strands Figure 6). The circular phage DNA is thereby integrated into the host genome as a linear prophage. Comparison of the phage sequences to the genome sequence of *A. baumannii* AbPK1 revealed that the attachment site sequence is a 22-bp sequence 5’-AAAAAGCGCTCAATCTAGAGCG-3’, located in the phage genome upstream of the predicted tyrosine family integrase encoding gene (Figure 4). This sequence was described as target site duplication (TSD) in the *A. baumannii* AbPK1 genome [60]. Identical *attB* sites were also identified from the lysogenic strains #5707 and #5920.

To provide experimental evidence of the temperate nature of the phages, phage fEg-Aba01 resistant derivatives, i.e., potential lysogens, of *A. baumannii* strain #6597 were isolated from fEg-Aba01 –infected bacterial lawns. PCR using phage-specific and *att-*site flanking primers (Table S2, Figure S2) was used to detect the presence of prophage in the lysogen candidates (Table S5). All the candidates proved to be positive confirming the temperate nature of the phage.

## 4. Discussion

We present here our findings suggesting that the three isolated *A. baumannii* siphophages were very likely prophages induced spontaneously during the culture of the lysogenic strains #5707 and #5920. As there is only limited information of the biological properties of temperate *A. baumannii* phages we present here a detailed characterization of the phages, and show experimentally that the prophages in #5707 and #5920 can be induced, and on the other hand that the phages can lysogenize a non-lysogenic strain. This indicated that the phages are fully functional. It is worth noting that these two lysogenic strains, in addition to sharing their MLST and capsule types, carry almost identical prophages, therefore either of them or both could be the original host of the isolated temperate phages. Comparison of the phage and prophage sequences demonstrated that they were almost 100% identical differing only by a few occurrences of substitutions/deletions/insertions (Figure S5). It is, therefore, very likely that some of the mutations took place during the laboratory propagation of the phages as none of the three were fully identical to the prophage sequences in strains #5707 and #5920 (Figure S5). Based on the whole-genome VICTOR phylogenetic prediction (Figure S5A) it is apparent that fLi-Aba02 is closest to the prophages of #5707 and #5920, followed by fLi-Aba03, leaving fEg-Aba01 as most distant. Of special interest is the deletion/gene fusion in fEg-Aba01 (RoD-02, Table 3), that potentially could affect the phage biology. The deleted sequence carries the gene predicted to encode a peptidoglycan hydrolase (*g19* of fLi-Aba02 and fLi-Aba03) that might be involved in the lysis of the bacterial cell and the release of the phage progeny. This deletion seems to be well tolerated as we observed no gross differences between the three phages. The same applied apparently to the other major differences in fEg-Aba01 affected genes *g48* and *g28c* (RoD-05 and RoD-07), truncating both from the 3’-end. This short-term evolution of the phages allows identification of non-essential regions of the phage genomes.

When isolating the phages, among the 20 clinical *A. baumannii* strains was one, #6597 that was sensitive to the phages and made their detection possible. Interestingly, among the 53 *Acinetobacter* strains representing 7 species in addition to *A. baumannii,* only two *A. baumannii* strains were sensitive to the phages. This narrow host range could be due to several different reasons: (i) the phage receptor structures (LOS, capsule or outer membrane proteins) of the strains are different, (ii) the strains might be lysogens similar to #5707 and #5920, (iii) other resident prophages may encode an infection exclusion system or (iv) the presence of phage-specific spacers in the CRISPR/Cas locus [76]. The fact that all the strains shared the LOS type, and that the phage-sensitive strain #6597 represents capsule type KL3 (Table 4) makes it likely that the phages are KL3 specific. There is a discrepancy; the temperate phages infect only the KL3 strain but reside as prophages in KL25 strain. The structures of the KL3 and KL25 capsule polysaccharides are completely different [77,78], thus, one can ask the question how they entered in the first place into the KL25 strain. One can entertain two scenarios. First, if the phage receptor is indeed KL3, then the prophage should have entered the KL25 lineage before they have taken up the KL25 capsule gene cluster, or alternatively, the prophage has been transduced by a KL25 specific phage, or second, the phage receptor is not the KL3 capsule but an outer membrane protein. The fact that the phage plaque is surrounded by a halo, indicates that a diffusible enzymatic activity, likely associated with the phage tail, is degrading the capsular polysaccharide around the plaque, indirectly indicating that the KL3 capsule is specifically recognized by the phage. This question remains to be addressed in future.

The phages were morphologically typical siphoviruses with heads of about 60 nm in diameter, and flexible non-contractile tails of 140–150 nm in length, the genomes 33.8–35.0 kb in size encoding 52–54 predicted genes. We identified the *attP* sequence of the phage, and demonstrated experimentally that the phage has cohesive termini that the PhageTerm tool predicted to be 10 nt long (Figure S3). The adsorption curves of the phages (Figure 2) demonstrated that the phages adsorb to the bacteria extremely rapidly and efficiently with >95% of phages adsorbed already at the first time point of 1 min.

Several genomic data screening studies have been carried out to identify prophages that indeed turn out to be very abundant in *A. baumannii* genomes [33,61,79]. It is apparent that many of the identified prophages may also be involved in horizontal gene transfer and may carry genes encoding antibiotic resistance or virulence factors [79], however, this was not the case with the prophages identified in this work. Of note, the gene claimed to encode sialic acid acetyltransferase 3 in GI5 of AbPK1 [60] was here based on HHpred search annotated as acetylxylan esterase like protein (Gp19), thus a hydrolase, so it very likely may have a function in capsule degradation. While spontaneous induction of the prophages as described in the present study has not been reported earlier, prophage induction using mitomycin C has been reported [33,79]. Similarly, the phage vB_AbaS_TRS1, a 40.7 kb siphovirus, was induced by mitomycin C treatment from the *A. baumannii* strain A118 culture [80], however, it did not show any similarity to fEg-Aba01 and the other phages described here. On the other hand, based on the >99% identity of prophage GI5 of AbPK1 is very likely that it would behave identically to the spontaneously inducible phages described in this work.

As temperate phages are not considered suitable for phage therapy, non-lysogenic derivatives of the isolated phages could be constructed in future by targeted deletion of the lysogen decision gene cluster, similar to the strategy used in the recent *Mycobacterium abscessus* phage treatment case [81], provided that the selected phages do not carry any harmful genes. This could be worthwhile if there are difficulties in isolating *Acinetobacter* phages that we have experienced in Finland, and as the adsorption curves of the phages showed extraordinary adsorption rates suggesting also high efficiency in killing the target bacteria [82].

## Figures and Tables

**Table 1:** Phage particle and genome overview. Phage dimensions were measured by TEM and represent mean values for at least 10 measured phage particles.

Bacteriophage	fEg-Aba01	fLi-Aba02	fLi-Aba03
Capsid size, nm	58.4 ± 4	63 ± 2	58.6 ± 5
Tail length, nm	156 ± 9	136 ± 1.8	142 ± 0.8
Tail width, nm	10.8 ± 0.3	9 ± 0.2	8.5 ± 0.9
Genome size, bp	33,779	35,053	34,931
GC content (%)	40.1	40.2	40.1
*n* genes	52	53	54
*n* tRNAs	1	1	1

**Table 2:** The lysogen decision gene cluster in phages fEg-Aba01, fLi-Aba02, and fLi-Aba-03.

Gene	Location	Predicted Function	Amino Acids
g25_fEg-Aba01_	20438-21397	Integrase	319
g27_fLi-Aba02_	22436-21477		319
g27_fLi-Aba03_	22436-21477		319
g26c_fEg-Aba01_	21614-21363	Excisionase	83
g28c_fLi-Aba02_	22653-22402		83
g28c_fLi-Aba03_	22653-22402		83
g27c_fEg-Aba01_	22353-21667	Arginine repressor	228
g29c_fLi-Aba02_	23392-22706		228
g29c_fLi-Aba03_	23392-22706		228
g28c_fEg-Aba01_	23182-22595	ADP-ribosyltransferase	195
g30c_fLi-Aba02_	24429-23392		345
g30c_fLi-Aba03_	24267-23392		291
g29c_fEg-Aba01_	23494-23258	Antitoxin	78
g31c_fLi-Aba02_	24741-24505		78
g31c_fLi-Aba03_	24579-24343		78
g30c_fEg-Aba01_	24113-23478	Xre family transcriptional regulator	211
g32c_fLi-Aba02_	25360-24725		211
g32c_fLi-Aba03_	25198-24563		211
g31c_fEg-Aba01_	24406-24116	Hypothetical protein	96
g33c_fLi-Aba02_	25653-25363		96
g33c_fLi-Aba03_	25491-25201		96
g32c_fEg-Aba01_	25397-24657	Repressor protein CI	246
g34c_fLi-Aba02_	26596-25904		230
g34c_fLi-Aba03_	26434-25742		230
g33_fEg-Aba01_	25663-25421	Cro repressor	80
g35_fLi-Aba02_	26910-26725		61
g35_fLi-Aba03_	26748-26521		75

**Table 3:** The regions of differences (RoD) between the nucleotide sequences of the three temperate *Acinetobacter* phages and the prophage loci of *A. baumannii* strains AbPK1, #5707 and #5920. See Figure S5 for details.

Region of Difference	Location in fEg-Aba01	Description
RoD-01	18065..18066	A 207 bp in-frame deletion in *g20* of fEg-Aba01 that causes a 69 amino acid truncation of Gp20.
RoD-02	13550..13551	A 832 bp deletion in fEg-Aba01 that generates a fusion of genes corresponding to *g18* and *g20* of phages fLi-Aba02 and fLi-Aba03. The deletion includes the gene *g19* of the latter.
RoD-03	6915..6925	Five nucleotide substitutions in a stretch of 11 bp within *g10* of AbPK1 prophage causing one (Arg-to-Lys) neutral substitution.
RoD-04	32618	A silent one nucleotide substitution in *g50* of fEg-Aba01
RoD-05	32453..32454	A 67 bp deletion in *g50* of fEg-Aba01 causing a frame shift mutation and truncating the 3’-end of *g48*
RoD-06	24177	A one bp substitution in gene *g32c* causing a Ser to Phe substitution in the AbPK1 protein
RoD-07	22613..22772	A stretch of three variants of 27 bp repetitions (6–13 repetitions) in the 3’end of gene *g28c*. Also out of frame deletions in the fEg-Aba01 gene causing frame shift and 3’-end truncation. Identical full length region is present in fLi-Aba02, and the #5707 and #5920 prophages
RoD-08	21103	A silent one bp substitution in the integrase gene *g27* of AbPK1 prophage

**Table 4:** Whole genome sequence data based overview of *A. baumannii* strains.

Strain	MLST	LOS-Type	K-Type	Prophage
#5707	1114, 1841	OCL1	KL25	Yes
#5907	1806, 208	OCL1	KL2	Partial
#5920	1114, 1841	OCL1	KL25	Yes
#6597	1816, 195	OCL1	KL3	No
AbPK1	452	OCL1	KL3	Yes

## Supplementary Materials

The following are available online at https://www.mdpi.com/1999-4915/12/6/604/s1, Figure S1: plaque morphology of fEg-Aba01. Figure S2: schematic representation of the primers used to confirm the presence of the prophage in bacterial genomes. B, B’, P and P’ represent the bacterial and phage attachment site sequences. Figure S3: restriction digestion analysis of phages fEg-Aba01 and fLi-Aba02 digested with 8 different enzymes demonstrating the >99% sequence identity of the phages. Figure S4: PhageTerm analysis of the fEg-Aba01 sequence read data demonstrating the presence of HK97 type 10 nt long 3’ cohesive ends. Figure S5: comparison of the of the nucleotide sequences of the three temperate *Acinetobacter* phages and the prophage loci of *A. baumannii* strains AbPK1, #5707 and #5920. Figure S6: BLASTN alignment of the prophage of *A. baumannii* strain AbPK1 against the whole genome de novo assembly contigs of strains #5707, #5907, #5920, and #6597. The ABPK1 sequence contained short prophage-flanking sequences up- and down-stream of the prophage. While #6597 completely lacks the prophage, it is completely present in #5707 and #5920, and partially in #5907. Table S1: bacterial strains used in the work. Table S2: oligonucleotide primers used for PCR in the work. Table S3: annotations and comparisons of the phage and prophage gene products. Table S4: detection of prophage specific sequences by PCR in the clinical strains used for phage enrichment using prophage-specific primer pairs. Table S5: detection of prophage sequences in lysogens by PCR.
