# Supplementary material for: Identification and Functional Analysis of Temperate Siphoviridae Bacteriophages of Acinetobacter baumannii
Source: Viruses. 2020 May 31;12(6):604. doi: 10.3390/v12060604 (PMC7354433; doi:10.3390/v12060604)
Supplement: Supplementary file 1 [file viruses-12-00604-s001.pdf]

Supplementary data

Identification and functional analysis of of temperate siphoviridae bacteriophages of *Acinetobacter baumannii*

Shimaa Badawy[1,2], Maria I. Pajunen[1], Johanna Haiko[3], Zakaria A. M. Baka[2], Mohamed I. Abou-Dobara[2], Ahmed K. A. El-Sayed[2], Mikael Skurnik[1,3,*]

[1] Department of Bacteriology and Immunology, Medicum, Human Microbiome Research Program, Faculty of Medicine, University of Helsinki, Helsinki, Finland
[2] Department of Botany and Microbiology, Faculty of Science, Damietta University, New Damietta, Egypt
[3] Division of Clinical Microbiology, Helsinki University Hospital, HUSLAB, Helsinki, Finland.
[*] Corresponding author: Mikael Skurnik, e-mail: mikael.skurnik@helsinki.fi. Tel.: +358 2941 26464

Supplementary figures S1 – S5

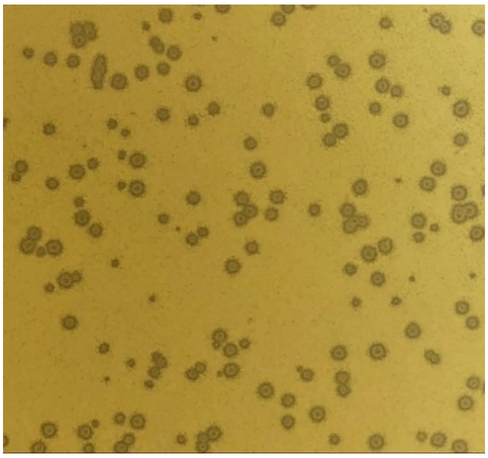

Figure S1. Plaque morphology of fEg-Aba01.

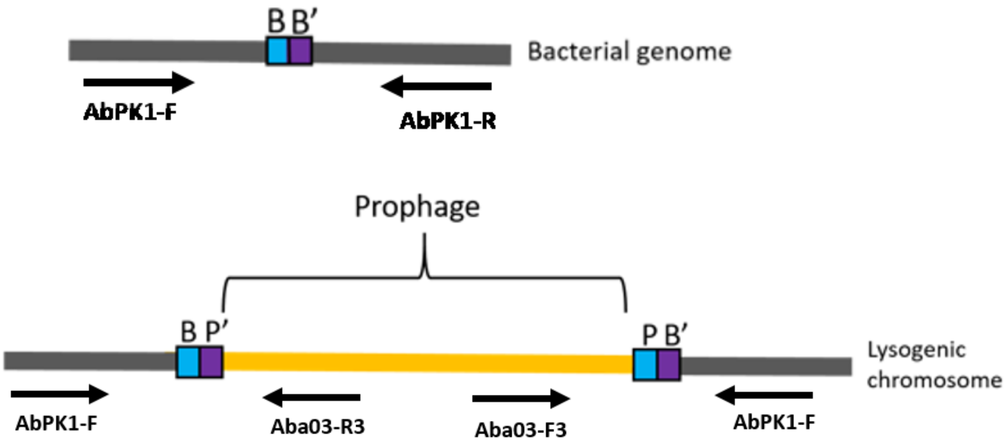

Figure S2. Schematic representation of the primers used to confirm the presence of the prophage in bacterial genomes. B, B′, P and P′ represent the bacterial and phage attachment site sequences.

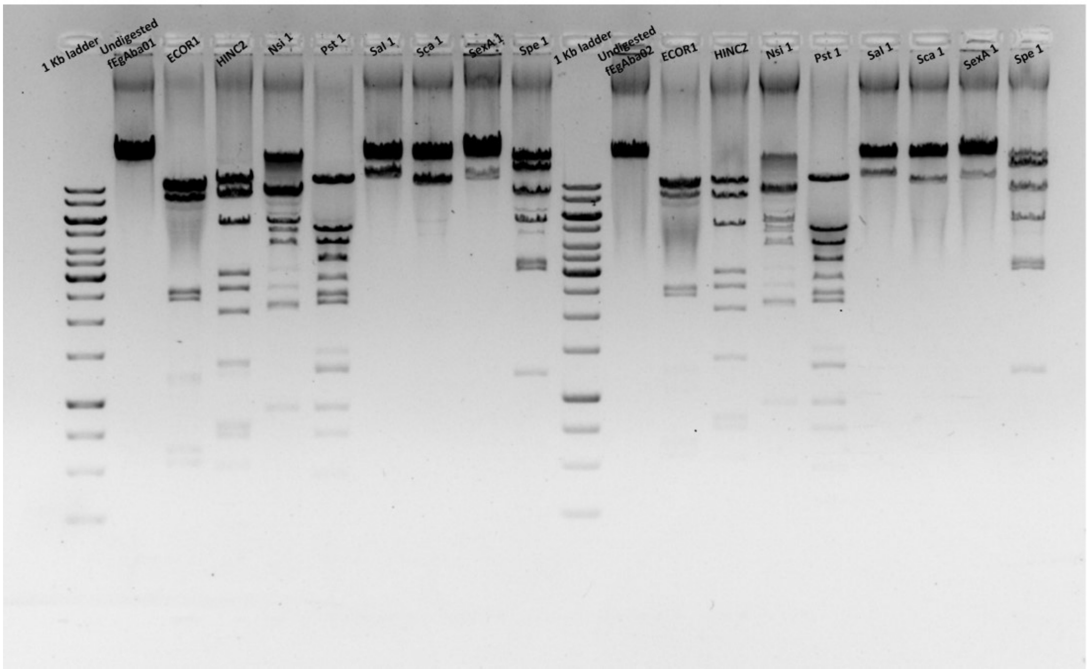

Figure S3. Restriction digestion analysis of phages fEg-Aba01 and fLi-Aba02 digested with 8 different enzymes demonstrating the >99% sequence identity of the phages

fEg_Ab01 PhageTerm Analysis

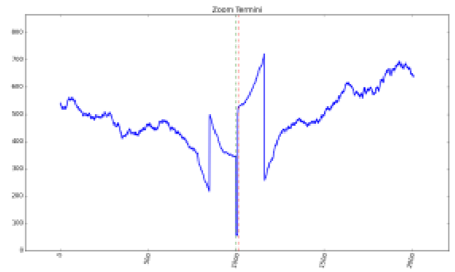

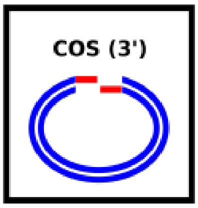

PhageTerm Method

Ends	Left (red)	Right (green)	Permuted	Orientation	Class	Type
Non Red.	20230	20219	No	NA	COS (3')	HK97

Sequence cohesive: CGCCCCCCAT

Strand	Location	T	pvalue
+	20230	0.95	8.32e-158
	21651	0.05	1.00e+00
	10519	0.05	1.00e+00
	1856	0.04	5.18e-01
	20232	0.04	3.95e-05
-	20219	0.90	2.42e-101
	9160	0.05	2.16e-01
	28505	0.04	1.00e+00
	26697	0.04	1.00e+00
	1690	0.04	1.00e+00

T (Start. Pos. Cov. / Whole Cov.)

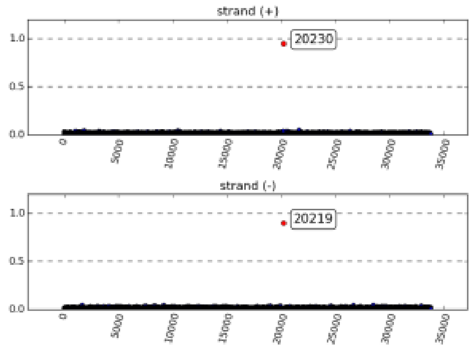

Figure S4. PhageTerm analysis of the fEg-Aba01 sequence read data demonstrating the presence of HK97 type 10 nt long 3′cohesive ends.

Figure S5. Comparison of the of the nucleotide sequences of the three temperate *Acinetobacter* phages and the prophage loci of *A. baumannii* strains AbPK1, #5707 and #5920. The relationship analysed by Victor (**A**). CLUSTAL format alignment (**B**). In the Clusta alignment the *attB / attP* sequences and the 3'-cohesive end sequence are highlighted in green. The locations with differences between the sequences are highlighted in yellow. The numbering of the bold upper case nucleotides refers to that of fEg-Aba01 (acc. no. MT344103). To make the file more manageable, the regions with 100% identity between all six sequences have been removed indicated by gray-shaded blocks. The eight regions of difference (RoD) have been labelled serially as RoD-01 – RoD-08. Note that the sequences are presented in the prophage state as in the genome of AbPK1 (acc. no. CP024576.1).

A

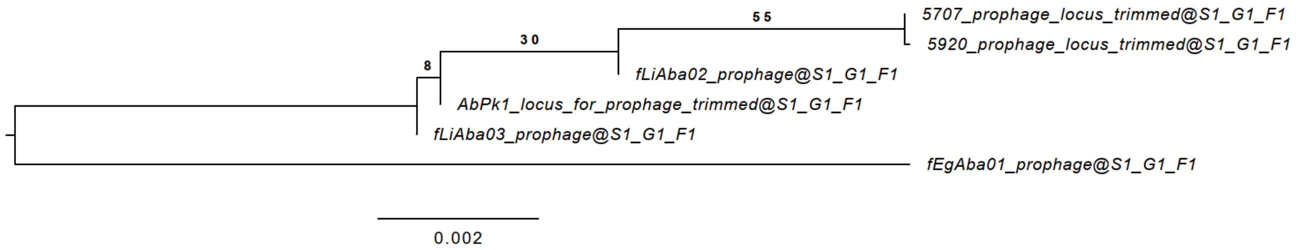

B

```
                    20301     attB/attP                                   20242
AbPk1               aaaaagcgctcaatctagagcggttgttgcctatataaatcagtaggttaaataaaattg
fEg-Aba01           AaaaagcgctcaatctagagcggttgttgcctatataaatcagtaggttaaataaaattG
fLi-Aba02           aaaaagcgctcaatctagagcggttgttgcctatataaatcagtaggttaaataaaattg
fLi-Aba03           aaaaagcgctcaatctagagcggttgttgcctatataaatcagtaggttaaataaaattg
#5707               aaaaagcgctcaatctagagcggttgttgcctatataaatcagtaggttaaataaaattg
#5920               aaaaagcgctcaatctagagcggttgttgcctatataaatcagtaggttaaataaaattg
                    ************************************************************
//
Δ(20241..18140)
//
                    18141                                                  18082
AbPk1               tcccttcccggccgaagcaaagcccgctaaaccactactcgttgtagaattgctgggtac
fEg-Aba01           TcccttcccggccgaagcaaagcccgctaaaccactactcgttgtagaattgctgggtaC
fLi-Aba02           tcccttcccggccgaagcaaagcccgctaaaccactactcgttgtagaattgctgggtac
fLi-Aba03           tcccttcccggccgaagcaaagcccgctaaaccactactcgttgtagaattgctgggtac
#5707               tcccttcccggccgaagcaaagcccgctaaaccactactcgttgtagaattgctgggtac
#5920               tcccttcccggccgaagcaaagcccgctaaaccactactcgttgtagaattgctgggtac
                    ************************************************************

                                   18066 RoD-01
AbPk1               taagtttcctaataaaatagctaattgggtgttattgcatttcacaactgctgtaagcgt
fEg-Aba01           taagtttcctaataaA--------------------------------------------
fLi-Aba02           taagtttcctaataaaatagctaattgggtgttattgcatttcacaactgctgtaagcgt
fLi-Aba03           taagtttcctaataaaatagctaattgggtgttattgcatttcacaactgctgtaagcgt
#5707               taagtttcctaataaaatagctaattgggtgttattgcatttcacaactgctgtaagcgt
#5920               taagtttcctaataaaatagctaattgggtgttattgcatttcacaactgctgtaagcgt
                    ****************

AbPk1               tacactttgtgctgatgtatcaattaaatcacttacaagagcattacctacggcagttga
fEg-Aba01           ------------------------------------------------------------
fLi-Aba02           tacactttgtgctgatgtatcaattaaatcacttacaagagcattacctacggcagttga
fLi-Aba03           tacactttgtgctgatgtatcaattaaatcacttacaagagcattacctacggcagttga
#5707               tacactttgtgctgatgtatcaattaaatcacttacaagagcattacctacggcagttga
#5920               tacactttgtgctgatgtatcaattaaatcacttacaagagcattacctacggcagttga
```

```
AbPk1         gattgttactcccgattcagagtaaatgggttgcactgtagcaccactttgcaatgctaa
fEg-Aba01     ------------------------------------------------------------
fLi-Aba02     gattgttactcccgattcagagtaaatgggttgcactgtagcaccactttgcaatgctaa
fLi-Aba03     gattgttactcccgattcagagtaaatgggttgcactgtagcaccactttgcaatgctaa
#5707         gattgttactcccgattcagagtaaatgggttgcactgtagcaccactttgcaatgctaa
#5920         gattgttactcccgattcagagtaaatgggttgcactgtagcaccactttgcaatgctaa

                                                         18065            18049
AbPk1         aagtctattattcactttatccgtcaatgaagccgatgtgttttcaaaaacccaatgccc
fEg-Aba01     -------------------------------------------TcaaaaacccaatgccC
fLi-Aba02     aagtctattattcactttatccgtcaatgaagccgatgtgttttcaaaaacccaatgccc
fLi-Aba03     aagtctattattcactttatccgtcaatgaagccgatgtgttttcaaaaacccaatgccc
#5707         aagtctattattcactttatccgtcaatgaagccgatgtgttttcaaaaacccaatgccc
#5920         aagtctattattcactttatccgtcaatgaagccgatgtgttttcaaaaacccaatgccc
                                                         *****************

//
Δ(18048..13609)
//

              13608                                                  13551
AbPk1         atcaaatcgggatatgatcgcaatcttccgaaaatatctggaatacggccattgatacga
fEg-Aba01     AtcaaatcgggatatgatcgcaatcttccgaaaatatctggaatacggccattgataC--
fLi-Aba02     atcaaatcgggatatgatcgcaatcttccgaaaatatctggaatacggccattgatacga
fLi-Aba03     atcaaatcgggatatgatcgcaatcttccgaaaatatctggaatacggccattgatacga
#5707         atcaaatcgggatatgatcgcaatcttccgaaaatatctggaatacggccattgatacga
#5920         atcaaatcgggatatgatcgcaatcttccgaaaatatctggaatacggccattgatacga
              **********************************************************

              RoD-02
AbPk1         ggttgatttgatcgttgagctaattcgttgtttgcagatccggccgttggggacggcggt
fEg-Aba01     ------------------------------------------------------------
fLi-Aba02     ggttgatttgatcgttgagctaattcgttgtttgcagatccggccgttggggacggcggt
fLi-Aba03     ggttgatttgatcgttgagctaattcgttgtttgcagatccggccgttggggacggcggt
#5707         ggttgatttgatcgttgagctaattcgttgtttgcagatccggccgttggggacggcggt
#5920         ggttgatttgatcgttgagctaattcgttgtttgcagatccggccgttggggacggcggt

AbPk1         ttaggcatagttagaactgtataaagtgtgtatgctgttgtaattgcaacaattgcatag
fEg-Aba01     ------------------------------------------------------------
fLi-Aba02     ttaggcatagttagaactgtataaagtgtgtatgctgttgtaattgcaacaattgcatag
fLi-Aba03     ttaggcatagttagaactgtataaagtgtgtatgctgttgtaattgcaacaattgcatag
#5707         ttaggcatagttagaactgtataaagtgtgtatgctgttgtaattgcaacaattgcatag
#5920         ttaggcatagttagaactgtataaagtgtgtatgctgttgtaattgcaacaattgcatag

AbPk1         taaatccacattgcaatagagattggctcctgtggcatgactacaacataaaataccccc
fEg-Aba01     ------------------------------------------------------------
fLi-Aba02     taaatccacattgcaatagagattggctcctgtggcatgactacaacataaaataccccc
fLi-Aba03     taaatccacattgcaatagagattggctcctgtggcatgactacaacataaaataccccc
#5707         taaatccacattgcaatagagattggctcctgtggcatgactacaacataaaataccccc
#5920         taaatccacattgcaatagagattggctcctgtggcatgactacaacataaaataccccc

AbPk1         tctaatttttgaagcttttcaatatccctcttatttttaggggtaatattttgagactca
fEg-Aba01     ------------------------------------------------------------
fLi-Aba02     tctaatttttgaagcttttcaatatccctcttatttttaggggtaatattttgagactca
fLi-Aba03     tctaatttttgaagcttttcaatatccctcttatttttaggggtaatattttgagactca
#5707         tctaatttttgaagcttttcaatatccctcttatttttaggggtaatattttgagactca
#5920         tctaatttttgaagcttttcaatatccctcttatttttaggggtaatattttgagactca

AbPk1         gcaattgcaccatgatatatttttgtgccaccgggtaggctaggagcaaaatgttgagct
fEg-Aba01     ------------------------------------------------------------
fLi-Aba02     gcaattgcaccatgatatatttttgtgccaccgggtaggctaggagcaaaatgttgagct
fLi-Aba03     gcaattgcaccatgatatatttttgtgccaccgggtaggctaggagcaaaatgttgagct
#5707         gcaattgcaccatgatatatttttgtgccaccgggtaggctaggagcaaaatgttgagct
#5920         gcaattgcaccatgatatatttttgtgccaccgggtaggctaggagcaaaatgttgagct
```

```
AbPk1          aagtaagcgcaaacatcctctacatcggcatatgcccatgtgtctcgtttatacacatca
fEg-Aba01      ------------------------------------------------------------
fLi-Aba02      aagtaagcgcaaacatcctctacatcggcatatgcccatgtgtctcgtttatacacatca
fLi-Aba03      aagtaagcgcaaacatcctctacatcggcatatgcccatgtgtctcgtttatacacatca
#5707          aagtaagcgcaaacatcctctacatcggcatatgcccatgtgtctcgtttatacacatca
#5920          aagtaagcgcaaacatcctctacatcggcatatgcccatgtgtctcgtttatacacatca

AbPk1          ggaacaatgacaactcgcttcaagctcatgtgtaaaacctcgtttccagaaaatgcatgc
fEg-Aba01      ------------------------------------------------------------
fLi-Aba02      ggaacaatgacaactcgcttcaagctcatgtgtaaaacctcgtttccagaaaatgcatgc
fLi-Aba03      ggaacaatgacaactcgcttcaagctcatgtgtaaaacctcgtttccagaaaatgcatgc
#5707          ggaacaatgacaactcgcttcaagctcatgtgtaaaacctcgtttccagaaaatgcatgc
#5920          ggaacaatgacaactcgcttcaagctcatgtgtaaaacctcgtttccagaaaatgcatgc

AbPk1          taacgacttcaagcggaatgaattgcgcaccgcgaagtgagagatgcaaaagttttccgc
fEg-Aba01      ------------------------------------------------------------
fLi-Aba02      taacgacttcaagcggaatgaattgcgcaccgcgaagtgagagatgcaaaagttttccgc
fLi-Aba03      taacgacttcaagcggaatgaattgcgcaccgcgaagtgagagatgcaaaagttttccgc
#5707          taacgacttcaagcggaatgaattgcgcaccgcgaagtgagagatgcaaaagttttccgc
#5920          taacgacttcaagcggaatgaattgcgcaccgcgaagtgagagatgcaaaagttttccgc

AbPk1          aataaaaaagcccgacatgtgtcgagcttttgggtccgtttgtaaaaaaaactatgcaag
fEg-Aba01      ------------------------------------------------------------
fLi-Aba02      aataaaaaagcccgacatgtgtcgagcttttgggtccgtttgtaaaaaaaactatgcaag
fLi-Aba03      aataaaaaagcccgacatgtgtcgagcttttgggtccgtttgtaaaaaaaactatgcaag
#5707          aataaaaaagcccgacatgtgtcgagcttttgggtccgtttgtaaaaaaaactatgcaag
#5920          aataaaaaagcccgacatgtgtcgagcttttgggtccgtttgtaaaaaaaactatgcaag

AbPk1          gtgaaataggctcatccaaagatttaaattccccctctccatttaaaaatttttttaaac
fEg-Aba01      ------------------------------------------------------------
fLi-Aba02      gtgaaataggctcatccaaagatttaaattccccctctccatttaaaaatttttttaaac
fLi-Aba03      gtgaaataggctcatccaaagatttaaattccccctctccatttaaaaatttttttaaac
#5707          gtgaaataggctcatccaaagatttaaattccccctctccatttaaaaatttttttaaac
#5920          gtgaaataggctcatccaaagatttaaattccccctctccatttaaaaatttttttaaac

AbPk1          gtgaagttaaatcttctcctgtaatatctagccaagcctcacatgcaaactcattacatg
fEg-Aba01      ------------------------------------------------------------
fLi-Aba02      gtgaagttaaatcttctcctgtaatatctagccaagcctcacatgcaaactcattacatg
fLi-Aba03      gtgaagttaaatcttctcctgtaatatctagccaagcctcacatgcaaactcattacatg
#5707          gtgaagttaaatcttctcctgtaatatctagccaagcctcacatgcaaactcattacatg
#5920          gtgaagttaaatcttctcctgtaatatctagccaagcctcacatgcaaactcattacatg

AbPk1          tgtaatcttccttccaagtccgtgataataggtgattgatattcatattaagccgcgcaa
fEg-Aba01      ------------------------------------------------------------
fLi-Aba02      tgtaatcttccttccaagtccgtgataataggtgattgatattcatattaagccgcgcaa
fLi-Aba03      tgtaatcttccttccaagtccgtgataataggtgattgatattcatattaagccgcgcaa
#5707          tgtaatcttccttccaagtccgtgataataggtgattgatattcatattaagccgcgcaa
#5920          tgtaatcttccttccaagtccgtgataataggtgattgatattcatattaagccgcgcaa

AbPk1          taaggggaatctttcaagatcgtaaatttcacctgttttaaccttgtttagctcaggtgc
fEg-Aba01      ------------------------------------------------------------
fLi-Aba02      taaggggaatctttcaagatcgtaaatttcacctgttttaaccttgtttagctcaggtgc
fLi-Aba03      taaggggaatctttcaagatcgtaaatttcacctgttttaaccttgtttagctcaggtgc
#5707          taaggggaatctttcaagatcgtaaatttcacctgttttaaccttgtttagctcaggtgc
#5920          taaggggaatctttcaagatcgtaaatttcacctgttttaaccttgtttagctcaggtgc

                                                                 13550     13541
AbPk1          tacagcctcaaaagtgcagttgccagtatcatcttttgaaatacttgctacctctaaagt
fEg-Aba01      --------------------------------------------------CctctaaagT
fLi-Aba02      tacagcctcaaaagtgcagttgccagtatcatcttttgaaatacttgctacctctaaagt
fLi-Aba03      tacagcctcaaaagtgcagttgccagtatcatcttttgaaatacttgctacctctaaagt
#5707          tacagcctcaaaagtgcagttgccagtatcatcttttgaaatacttgctacctctaaagt
#5920          tacagcctcaaaagtgcagttgccagtatcatcttttgaaatacttgctacctctaaagt
                                                                 **********
```

```
//
Δ(13540..6941)
//
              6940              RoD-03                                         6881
AbPk1         tcttaaaaatggggttgctggttgcttacttgtacctaactcaaggaatcgccagtaaaa
fEg-Aba01     TcttaaaaatggggtggcgggctgtctacttgtacctaactcaaggaatcgccagtaaaA
fLi-Aba02     tcttaaaaatggggtggcgggctgtctacttgtacctaactcaaggaatcgccagtaaaa
fLi-Aba03     tcttaaaaatggggtggcgggctgtctacttgtacctaactcaaggaatcgccagtaaaa
#5707         tcttaaaaatggggtggcgggctgtctacttgtacctaactcaaggaatcgccagtaaaa
#5920         tcttaaaaatggggtggcgggctgtctacttgtacctaactcaaggaatcgccagtaaaa
              *************** ** **.**..**********************************
//
Δ(6880..101)
//
              100                                                          41
AbPk1         attttccattttctctgttaatgccattgctctacctttaacttgattttaactttttg
fEg-Aba01     AttttccattttctctgttaatgccattgctctacctttaacttgattttaacttttttG
fLi-Aba02     attttccattttctctgttaatgccattgctctacctttaacttgattttaactttttg
fLi-Aba03     attttccattttctctgttaatgccattgctctacctttaacttgattttaactttttg
#5707         attttccattttctctgttaatgccattgctctacctttaacttgattttaactttttg
#5920         attttccattttctctgttaatgccattgctctacctttaacttgattttaactttttg
              ************************************************************

                                                         33779
              40                                        1 3'-cohesive end    33760
AbPk1         ctttaactttttctgagagggaatttttttgtgcgtgcgatgggggcggtgtccaacg
fEg-Aba01     CtttaactttttctgagagggaatttttttgtgcgtgcGAtgggggcggtgtccaacG
fLi-Aba02     ctttaactttttctgagagggaatttttttgtgcgtgcgatgggggcggtgtccaacg
fLi-Aba03     ctttaactttttctgagagggaatttttttgtgcgtgcgatgggggcggtgtccaacg
#5707         ctttaactttttctgagagggaatttttttgtgcgtgcgatgggggcggtgtccaacg
#5920         ctttaactttttctgagagggaatttttttgtgcgtgcgatgggggcggtgtccaacg
              ************************************************************
//
Δ(33759..32620)
//
              32619 RoD-04                                                 32560
AbPk1         ggccttcttgctttgcaggtggtctttaatgaaggtattgaccaaaacttggtaatcctt
fEg-Aba01     GtccttcttgctttgcaggtggtctttaatgaaggtattgaccaaaacttggtaatcctT
fLi-Aba02     ggccttcttgctttgcaggtggtctttaatgaaggtattgaccaaaacttggtaatcctt
fLi-Aba03     ggccttcttgctttgcaggtggtctttaatgaaggtattgaccaaaacttggtaatcctt
#5707         ggccttcttgctttgcaggtggtctttaatgaaggtattgaccaaaacttggtaatcctt
#5920         ggccttcttgctttgcaggtggtctttaatgaaggtattgaccaaaacttggtaatcctt
              * **********************************************************

AbPk1         ttcaagttgacggcgcttaggtccatcgaatgcttgaataacatctttaaagttctgcaa
fEg-Aba01     ttcaagttgacggcgcttaggtccatcgaatgcttgaataacatctttaaagttctgcaa
fLi-Aba02     ttcaagttgacggcgcttaggtccatcgaatgcttgaataacatctttaaagttctgcaa
fLi-Aba03     ttcaagttgacggcgcttaggtccatcgaatgcttgaataacatctttaaagttctgcaa
#5707         ttcaagttgacggcgcttaggtccatcgaatgcttgaataacatctttaaagttctgcaa
#5920         ttcaagttgacggcgcttaggtccatcgaatgcttgaataacatctttaaagttctgcaa
              ************************************************************

              32499                                     32454 RoD-05
AbPk1         agccatgtacttttctgcagtctcttgccgaccctcaacatagatccgggcaagaaactt
fEg-Aba01     AgccatgtacttttctgcagtctcttgccgaccctcaacatagatC--------------
fLi-Aba02     agccatgtacttttctgcagtctcttgccgaccctcaacatagatccgggcaagaaactt
fLi-Aba03     agccatgtacttttctgcagtctcttgccgaccctcaacatagatccgggcaagaaactt
#5707         agccatgtacttttctgcagtctcttgccgaccctcaacatagatccgggcaagaaactt
#5920         agccatgtacttttctgcagtctcttgccgaccctcaacatagatccgggcaagaaactt
              **********************************************
```

```
                                                                     32453
AbPk1           tttcttaaaaagtaaatgaagatcatcctttgaattaccggtcttttgcctgatctgttc
fEg-Aba01       -----------------------------------------------------Tctgttc
fLi-Aba02       tttcttaaaaagtaaatgaagatcatcctttgaattaccggtcttttgcctgatctgttc
fLi-Aba03       tttcttaaaaagtaaatgaagatcatcctttgaattaccggtcttttgcctgatctgttc
#5707           tttcttaaaaagtaaatgaagatcatcctttgaattaccggtcttttgcctgatctgttc
#5920           tttcttaaaaagtaaatgaagatcatcctttgaattaccggtcttttgcctgatctgttc
                                                                     *******

//
Δ(32446..24227)
//

                24226                                           RoD-06     24167
AbPk1           agcttagagcaattcactggaattcttgagtacacaatgacacttcgatttggctactgg
fEg-Aba01       AgcttagagcaattcactggaattcttgagtacacaatgacacttcgatctggctactgG
fLi-Aba02       agcttagagcaattcactggaattcttgagtacacaatgacacttcgatctggctactgg
fLi-Aba03       agcttagagcaattcactggaattcttgagtacacaatgacacttcgatctggctactgg
#5707           agcttagagcaattcactggaattcttgagtacacaatgacacttcgatctggctactgg
#5920           agcttagagcaattcactggaattcttgagtacacaatgacacttcgatctggctactgg
                *************************************************.**********

//
Δ(24166..22787)
//
                                RoD-07
                22786         22772               22771                    22746
AbPk1           aaagaccatggtcaggctgttgctactggcgacaactcagcaagctctgctactggcgac
fEg-Aba01       AaagaccatggtcaG-------------------ActcagcaagctctgctactggcgaC
fLi-Aba02       aaagaccatggtcaggctgttgctactggcgacaactcagcaagctctgctactggcgac
fLi-Aba03       aaagaccatggtcaggctgttgctactggcgacaactcagcaagctctgctactggcgac
#5707           aaagaccatggtcaggctgttgctactggcgacaactcagcaagctctgctactggcgac
#5920           aaagaccatggtcaggctgttgctactggcgacaactcagcaagctctgctactggcgac
                ***************                   **************************

                22745                                                22691
AbPk1           aactcagcaagctctgctactggctacaactcagcaagctctgctactggcgacaactca
fEg-Aba01       AactcagcaagctctgctactggctacaaatcagcaagctctgctactggcgacA-----
fLi-Aba02       aactcagcaagctctgctactggctacaactcagcaagctctgctactggcgacaactca
fLi-Aba03       aactcagcaagctctgctactggckacaactcagcaagctctgctactggcgacaactca
#5707           aactcagcaagctctgctactggctacaactcagcaagctctgctactggcgacaactca
#5920           aactcagcaagctctgctactggctacaactcagcaagctctgctactggcgacaactca
                ************************ **** *************************

                                      22690
AbPk1           gcaagcttaacaactggacactactcagcaagctctgctactggcgacaactcagcaagc
fEg-Aba01       ----------------------Actcagcaagctctgctactggcgacaactcagcaagc
fLi-Aba02       gcaagcttaacaactggacactactcagcaagctctgctactggcgacaactcagcaagc
fLi-Aba03       gcaagcttaacaactggacactactcagcaagctctgctactggcgacaactcagcaagc
#5707           gcaagcttaacaactggacactactcagcaagctctgctactggcgacaactcagcaagc
#5920           gcaagcttaacaactggacactactcagcaagctctgctactggcgacaactcagcaagc
                                      **************************************

AbPk1           tctgctactggcgacaactcagcaagctctgctactggcgacaact--------------
fEg-Aba01       tctgctactggcgacaactcagcaagctctgctactggc---------------------
fLi-Aba02       tctgctactggctacaaatcagcaagctctgctactggcgacaactcagcaagctctgct
fLi-Aba03       tctgctactggckacaamtcagcaagctctgctactggcgacaact--------------
#5707           tctgctactggctacaaatcagcaagctctgctactggcgacaactcagcaagctctgct
#5920           tctgctactggctacaaatcagcaagctctgctactggcgacaactcagcaagctctgct
                ************ **** *********************

AbPk1           ------------------------------------------------------------
fEg-Aba01       ------------------------------------------------------------
fLi-Aba02       actggcgacaactcagcaagctctgctactggctacaaatcagcaagctctgctactggc
fLi-Aba03       ------------------------------------------------------------
#5707           actggcgacaactcagcaagctctgctactggctacaaatcagcaagctctgctactggc
#5920           actggcgacaactcagcaagctctgctactggctacaaatcagcaagctctgctactggc
```

```
AbPk1           ------------------------------------------------------------
fEg-Aba01       ------------------------------------------------------------
fLi-Aba02       gacaactcagcaagctctgctactggcgacaactcagcaagctctgctactggcgacaac
fLi-Aba03       ------------------------------------------------------------
#5707           gacaactcagcaagctctgctactggcgacaactcagcaagctctgctactggcgacaac
#5920           gacaactcagcaagctctgctactggcgacaactcagcaagctctgctactggcgacaac

                                     22613                                  22575
AbPk1           -cagcaagctctgctactggcaactgggcagcaagcttaacaaccgggcactattctgaa
fEg-Aba01       ---------------------AactgggcagcaagcttaacaaccgggcactattctgaA
fLi-Aba02       tcagcaagctctgctactggcaactgggcagcaagcttaacaaccgggcactattctgaa
fLi-Aba03       ----------------------------cagcaagcttaacaaccgggcactattctgaa
#5707           tcagcaagctctgctactggcaactgggcagcaagcttaacaaccgggcactattctgaa
#5920           tcagcaagctctgctactggcaactgggcagcaagcttaacaaccgggcactattctgaa
                                            ********************************
//
Δ(22574..21135)
//
                21134                          RoD-08                       21075
AbPk1           taaagatttcaaccgcttgggaacttaaaggtacttcccttgcacgaccatttttgtaa
fEg-Aba01       TaaagatttcaaccgcttgggaacttaaaggcacttcccttgcacgaccatttttgtaA
fLi-Aba02       taaagatttcaaccgcttgggaacttaaaggcacttcccttgcacgaccatttttgtaa
fLi-Aba03       taaagatttcaaccgcttgggaacttaaaggcacttcccttgcacgaccatttttgtaa
#5707           taaagatttcaaccgcttgggaacttaaaggcacttcccttgcacgaccatttttgtaa
#5920           taaagatttcaaccgcttgggaacttaaaggcacttcccttgcacgaccatttttgtaa
                *******************************.****************************
//
Δ(21074..20355)
//
                20354                                               20302
AbPk1           taagtcattgttttttataattattaaacaaatgaaatttgattgaatttaataaaaagc
fEg-Aba01       TaagtcattgttttttataattattaaacaaatgaaatttgattgaatttaaT-------
fLi-Aba02       taagtcattgttttttataattattaaacaaatgaaatttgattgaatttaat-------
fLi-Aba03       taagtcattgttttttataattattaaacaaatgaaatttgattgaatttaat-------
#5707           taagtcattgttttttataattattaaacaaatgaaatttgattgaatttaataaaaagc
#5920           taagtcattgttttttataattattaaacaaatgaaatttgattgaatttaataaaaagc
                *****************************************************
                 attB / attP
AbPk1           gctcaatctagagcgctcttcatatctgatttcttaagaagttttagtcgcatcaatatg
fEg-Aba01       ------------------------------------------------------------
fLi-Aba02       ------------------------------------------------------------
fLi-Aba03       ------------------------------------------------------------
#5707           gctcaatctagagcgctcttcatatctgatttcttaagaagttttagtcgcatcaatatg
#5920           gctcaatctagagcgctcttcatatctgatttcttaagaagttttagtcgcatcaatatg
```

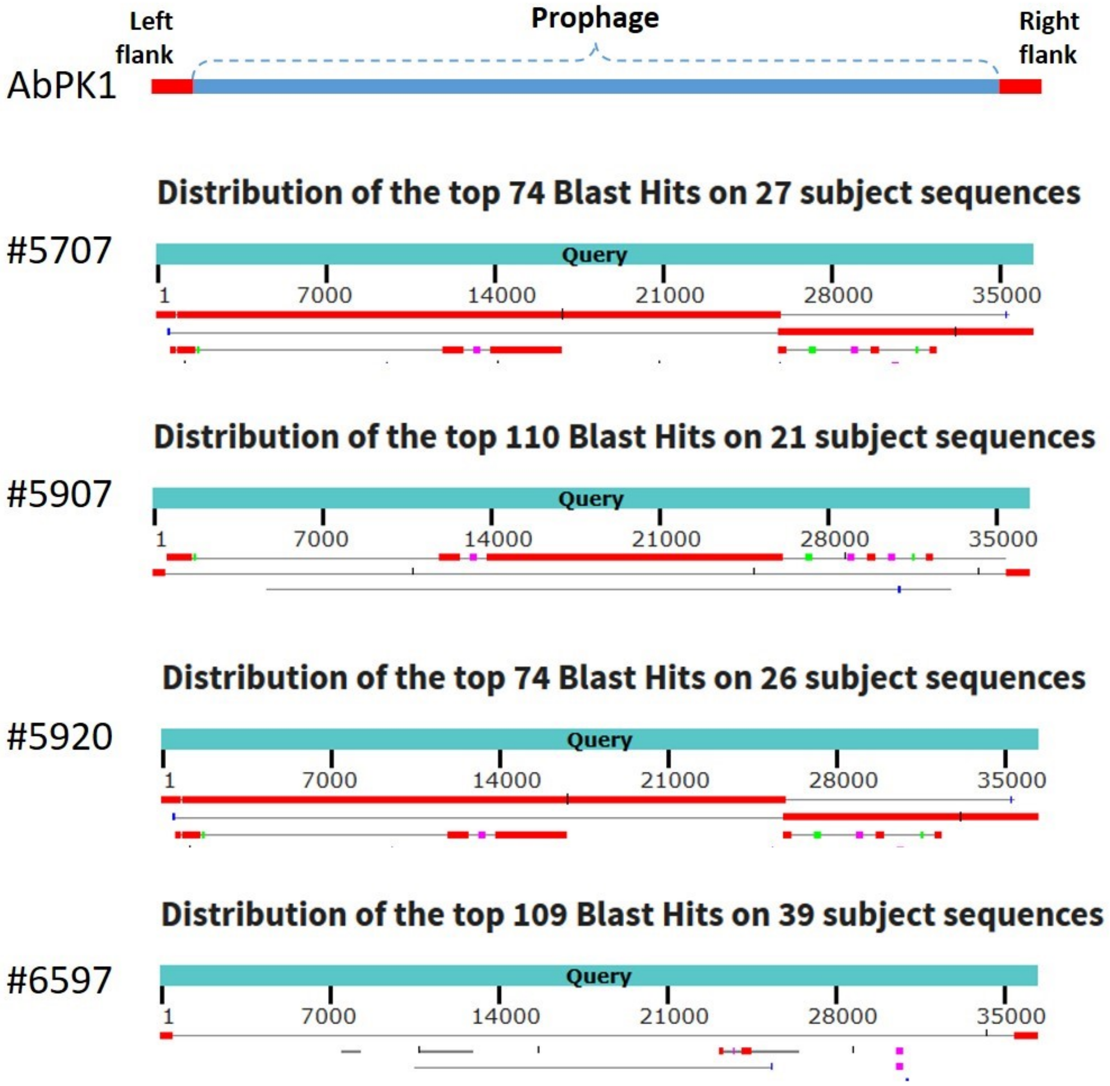

Figure S6. BLASTN alignment of the prophage of *A. baumannii* strain AbPK1 against the whole genome de novo assembly contigs of strains #5707, #5907, #5920, and #6597. The ABPK1 sequence contained short prophage-flanking sequences up- and down-stream of the prophage. While #6597 completely lacks the prophage, it is completely present in #5707 and #5920, and partially in #5907.

Supplementary tables S1 – S5.

Table S1. Bacterial strains used in the work.

SPECIES	STORAGE #	RESISTANCE PHENOTYPE, ORIGIN	SOURCE
Acinetobacter baumannii	5542	MDR, Not known	Huslab
	5568	Blood	Huslab
	5570	Blood	Huslab
	5596	MDR, bronchus	Huslab
	5597	MDR, rectum	Huslab
	5706	Tooth removal hole	Huslab
	5707	MDR, hip surgical wound	Huslab
	5729	MDR, rectum	Huslab
	5730	MDR, rectum	Huslab
	5731	Middle ear	Huslab
	5907	trachea	Huslab
	5910	MDR, skin	Huslab
	5911	MDR, rectal mucus	Huslab
	5919	MDR, rectal mucus	Huslab
	5920	MDR, Not known	Huslab
	5923	MDR, Wound pus	Huslab
	5924	Blood	Huslab
	5933	Urine	Huslab
	6594	Trachea	Huslab
	6597	MDR, Human sample, DSM 106838	DSMZ
	6898	MDR, Sacral chronic wound	Huslab
Acinetobacter calcoaceticus	5922	Blood	Huslab
Acinetobacter junii	5567	MDR, stool	Huslab
Acinetobacter lwoffii	5912	Blood	Huslab
	5921	Blood	Huslab
	5928	Blood	Huslab
Acinetobacter nosocomialis	5901	Foot surgical wound	Huslab
	5904	Peritoneal abscess	Huslab
	5929	Urine	Huslab
Acinetobacter pittii	5902	Not known	Huslab
	5903	Not known	Huslab
	5905	Leg wound pus	Huslab
	5906	Urine	Huslab
	5908	CSF	Huslab
	5909	Blood	Huslab
	5565	Blood	Huslab

Viruses

	5566	Blood	Huslab
	5673	Blood	Huslab
	5674	Wound pus	Huslab
	5930	Urine	Huslab
	5931	Urine	Huslab
	5573	Blood	Huslab
	5728	Wound pus	Huslab
	5914	Blood	Huslab
	5917	Blood	Huslab
	5918	Blood	Huslab
	5925	Blood	Huslab
Acinetobacter radioresistens	5915	Blood	Huslab
	5916	Blood	Huslab
Acinetobacter ursingii	5913	Blood	Huslab
	5927	Skin	Huslab
	5932	Urine	Huslab
Escherichia coli	5512	Blood	Huslab
	5517	Blood	Huslab
	5519	Blood	Huslab
	5520	Blood	Huslab
	5521	Blood	Huslab
	5522	Blood	Huslab
	5626	Urine	Huslab
	5627	Urine	Huslab
	5629	Abdominal punction fluid	Huslab
	5632	Urine	Huslab
	5633	Rectum mucus	Huslab
	5634	Rectum mucus	Huslab
	5636	Urine	Huslab
	5639	Urine	Huslab
	5760	ESBL, stool	Huslab
	5761	ESBL, blood	Huslab
	5762	ESBL, stool	Huslab
	5763	ESBL, urine	Huslab
	5764	ESBL, urine	Huslab
	5765	ESBL, urine	Huslab
	5766	Abscess in gluteal cleft	Huslab
	5767	Foot surgical wound	Huslab
	5769	ESBL	Huslab
	5510	Blood	Huslab
Klebsiella pneumoniae	5772	Urine	Huslab
Pseudomonas aeruginosa	5538	MDR, urine	Huslab
	5539	MDR, urine	Huslab

Pseudomonas monteilii/putida	5571	Blood	Huslab
Staphylococcus aureus	5696	MRSA, wound pus	Huslab
	5697	MRSA, vagina	Huslab
	5698	MRSA, pharynx	Huslab
	5699	MRSA, pharynx	Huslab
	5700	MRSA, pharynx	Huslab
	5701	MRSA, blood	Huslab
	5702	MRSA, pharynx	Huslab
	5703	MRSA, pharynx	Huslab
	5704	MRSA, pharynx	Huslab
	5705	MRSA, abscess in gluteal cleft	Huslab
Yersinia pseudotuberculosis	5926	Blood	Huslab

Table S2. Oligonucleotide primers used for PCR in the work.

Primer	Primer sequence 5' – 3'	Product size	Location CP024576.1	Annealing temp. °C	Purpose/ Comment
Aba03-F1<br>Aba03-R1	CGTAATGAAGTGCGGGATTT<br>ACCCTCTGACTCGCTCAAAA	700 bp	2549841<br>2549142	58	Prophage detection and confirmation of lysogeny
Aba03-F2<br>Aba03-R2	ATTAGGCACGCTTCACGTTT<br>CAAAGCTAAGGCATCCGAAG	404 bp	2565396<br>2564993	58	
Aba03-F3<br>AbPK1-R	GATAGCCATCGCTTGACCTC<br>GATGCAGGAGTGGATTACCG	1123 bp	2566646<br>2567768	55	Prophage *att* –site flanking primers used in different combinations to check the prophage status of *A. baumannii* strains. Primer pair AbPK1F/AbPK1-R produces a 905 bp PCR-product from a non-lysogen (Figure S2)
AbPK1-F<br>Aba03-R3	CGTGCAGTCATTGTCTTTCC<br>TGAAATTGAGGGCACAACTG	878 bp	2531906<br>2532783	55	
5707-F<br>5707-R	CGACCTTATCAAAGCCGGTA<br>TGTCACGGTTCCATCAATGT	416 bp	2532841<br>2532426	55	Prophage detection and confirmation of lysogeny

Table S3. Annotations and comparisons of the phage and prophage gene products. The identities of ther gene products are compoared to those of fEg-Aba01. The comments column contains information of discrepansies between the genomes.

**fEg-Aba01**				**fLi-Aba02**					**fLi-Aba03**					**Prophage of *A. baumannii* AbPK1** (Acc.no. CP024576.1)							**Functions predicted by BLASTP (Acc.no) and HHPRED (PDB ID) searches**					
**Gp**	**Genomic location**	**AA**	**MW**	**Gp**	**Genomic location**	**AA**	**MW**	**% ID**	**Gp**	**Genomic location**	**AA**	**MW**	**% ID**	**Gp**	**Phage genome location**	**Prophage genome location**	**Locus tag**	**AA**	**MW**	**% ID**	**Predicted function**	**Best hit**	**BLASTP e-value (identity %)**	**HHPRED e-value (probability)**	**Organism**	**Comments**
Gp01	73..555	160	17515	Gp01	73:555	160	17515	100	Gp01	73:555	160	17515	100	Gp01	73:555	c (2553031..2553513)	CTI08_12430	160	17515	100	Terminase, small subunit	3ZQP_I		5.6e-15 (99.44)	SPP1-like bacteriophage	
Gp02	575..766	63	7331	Gp02	575:766	63	7331	100	Gp02	575:766	63	7331	100	Gp02	575:766	c (2552820..2553011)	CTI08_12425	63	7331	100	Hypothetical protein	WP_001191044.1	5e-39 (100)		Acinetobacter baumannii	
Gp03	938..2632	564	62550	Gp03	938:2632	564	62550	100	Gp03	1040:2632	530	58706	100	Gp03	1040:2632	c (2550954..2552648)	CTI08_12420	530	58706	100	Terminase large subunit	4IDH		1.3e-29 (99.96)	Shigella phage Sf6	
Gp04	2629..3855	408	45765	Gp04	2629:3855	408	45765	100	Gp04	2629:3855	408	45765	100	Gp04	2629:3855	c (2549731..2550957)	CTI08_12415	408	45765	100	Phage portal protein	3KDR		4.6e-39 (100)	HK97 Family Phage	
Gp05	3848..4510	220	24426	Gp05	3848:4510	220	24426	100	Gp05	3848:4510	220	24426	100	Gp05	3848:4510	c (2549076..2549738)	CTI08_12410	220	24426	100	Capsid protein	106E		4.6e-15 (99.1)	Epstein-Barr virus (strain B95-8)	
Gp06	4503..5675	390	42375	Gp06	4503:5675	390	42375	100	Gp06	4503:5675	390	42375	100	Gp06	4503:5675	c (2547911..2549083)	CTI08_12405	390	42375	100	Major capsid protein	3QPR		1.7e-45 (100)	Enterobacteria phage HK97	
Gp07	5723..5899	58	6879	Gp07	5723:5899	58	6879	100	Gp07	5723:5899	58	6879	100	Gp07	5723:5899			58	6879	100	Hypothetical protein	YP_009593484.1	5e-07 (41.4)		Paracoccus phage Shpa	
Gp08	5896..6183	95	11236	Gp08	5896:6183	95	11236	100	Gp08	5896:6183	95	11236	100	Gp08	5896:6183	(2547403..2547690)	CTI08_12400	95	11236	100	Portal protein	3JVO_G		3.3e-19 (99.71)	Enterobacteria phage HK97	
Gp09	6185..6541	118	13396	Gp09	6185:6541	118	13396	100	Gp09	6185:6541	118	13396	100	Gp09	6185:6541	c (2547045..2547401)	CTI08_12395	118	13396	100	Head-tail adaptor	5A21_E		4.7e-20 (99.77)	BACILLUS PHAGE SPP1	
Gp10	6545..7030	161	18458	Gp10	6545:7030	161	18458	100	Gp10	6545:7030	161	18458	100	Gp10	6545:7030	c (2546556..2547041)	CTI08_12390	161	18430	99.38	Hypothetical protein	ASN69696.1	2e-38 (96)		Caudoviralis phage	
Gp11	7030..7404	124	13869	Gp11	7030:7404	124	13869	100	Gp11	7030:7404	124	13869	100	Gp11	7030:7404	c (2546182..2546556)	CTI08_12385	124	13869	100	Portal protein	5A21_G		2.2e-17 (99.62)	BACILLUS PHAGE SPP1	
Gp12	7474..7953	159	17629	Gp12	7474:7953	159	17629	100	Gp12	7474:7953	159	17629	100	Gp12	7474:7953	c (2545633..2546112)	CTI08_12380	159	17629	100	Major tail protein	2K4Q_A		3.7e-19 (99.74)	Enterobacteria phage lambda	
Gp13	7953..8369	138	15319	Gp13	7953:8369	138	15319	100	Gp13	7953:8369	138	15319	100	Gp13	7953:8369	c (2545217..2545633)	CTI08_12375	138	15319	100	Tail assembly chaperone	2OB9_B		7.7e-17 (99.5)	Enterobacteria phage HK97	
Gp14	8405..8620	71	8492	Gp14	8405:8620	71	8492	100	Gp14	8405:8620	71	8492	100	Gp14	8405:8620	c (2544966..2545181)	CTI08_12370	71	8492	100	Hypothetical protein	WP_000638276.1	1e-45 (100)		Acinetobacter baumannii	
Gp15	8659..9042	127	13832	Gp15	8659:9042	127	13832	100	Gp15	8659:9042	127	13832	100	Gp15	8659:9042	c (2544544..2544927)	CTI08_12365	127	13861	99.21	Hypothetical protein	2M4L_A		0.0000022 (98.08)	Bacteroides thetaiotaomicron	
Gp16	9102..12689	1195	131514	Gp16	9102:12689	1195	131514	100	Gp16	9102:12689	1195	131514	100	Gp16	9102:12689	c (2540897..2544484)	CTI08_12360	1195	131514	100	Tail length tape-measure protein	EXC34375.1	0.0 (95)		Acinetobacter sp. 869535	
Gp17	12691..13179	162	18285	Gp17	12691:13179	162	18285	100	Gp17	12691:13179	162	18285	100	Gp17	12691:13179	c (2540407..2540895)	CTI08_12355	162	18285	100	Hypothetical protein	WP_079378947.1	7e-117 (100)		Acinetobacter baumannii	
				Gp18	13172:13678	168	19238	100 (N-term)	Gp18	13172:13678	168	19238	100 (N-term)	Gp18	13172:13678	c (2539908..2540414)	CTI08_12350	168	19238	100 (N-term)	Hypothetical protein	WP_033502828.1	2e-121 (100)		Acinetobacter baumannii	C-terminus deleted in fEg-Aba01
				Gp19	13675:13995	106	12196		Gp19	13675:13995	106	12196		Gp19	13675:13995	c (2539591..2539911)	CTI08_12345	106	12196		Peptidoglycan hydrolase	3PBI_A		7.1e-13 (99.16%)	Mycobacterium tuberculosis	Deleted in fEg-Aba01
Gp18	13172..16018	948	106703	Gp20	13992:16850	952	106660	100 (C-term)	Gp20	13992:16850	952	106660	100 (C-term)	Gp20	13992:16850	c (2536736..2539594)	CTI08_12340	952	106660	100 (C-term)	Tail protein	YP_009289768.1	9e-149 (34)		Acinetobacter phage vB_AbaS_TRS1	Gp18 in fEg-Aba01 is a fusion between Gp18 (127 N-term aa) and Gp20 of fLi-Aba02
Gp19	16079..17941	620	68027	Gp21	16911:18773	620	68027	100	Gp21	16911:18773	620	68027	100	Gp21	16911:18773	c (2534813..2536561)	CTI08_12335	620	68027	100	Acetylxylan esterase like protein	1ZMB_D		8.4e-27 (99.92)	Clostridium acetobutylicum	
Gp20	17944..18516	190	20220	Gp22	18776:19555	259	27266	100	Gp22	18776:19555	259	27266	100	Gp22	18776:19555	c (2534031..2534810)	CTI08_12330	259	27266	100	Lectin-like protein	KCX86502.1	2e-92 (100)		Acinetobacter baumannii 15827	Gp20 of fEg-Aba01 has an internal deletion
Gp21	18564..18839	91	9954	Gp23	19603:19878	91	9954	100	Gp23	19603:19878	91	9954	100	Gp23	19603:19878	c (2533708..2533983)	CTI08_12325	91	9954	100	Holin	WP_032037508.1	2e-58 (99)		Acinetobacter sp. 742879	
Gp22	18823..19368	181	20144	Gp24	19862:20407	181	20144	100	Gp24	19862:20407	181	20144	100	Gp24	19862:20407	c (2533179..2533724)	CTI08_12320	181	20144	100	Lysozyme	3HDE_D		1.1e-35 (100)	Enterobacteria phage P21	
Gp23	19368..19736	122	14055	Gp25	20407:20775	122	14055	100	Gp25	20407:20775	122	14055	100	Gp25	20407:20775	c (2532672..2532866)	CTI08_12315	122	14055	100	Hypothetical protein	WP_088741716.1	2e-81 (100)		Acinetobacter baumannii	
Gp24	19972..20169	65	7470	Gp26	21011:21208	65	7470	100	Gp26	21011:21208	65	7470	100	Gp26	21011:21208	c (2532378..2532575)	CTI08_12305	65	7470	100	Hypothetical protein	WP_001186628.1	5e-40 (100)		Acinetobacter	
Gp25	20438..21397	319	37323	Gp27	21477:22436	319	37323	100	Gp27	21477:22436	319	37323	100	Gp27	21477:22436	c (2566108..2567067)	CTI08_12555	319	37323	100	Integrase	5C6K_A		1.3e-33 (100)	Enterobacteria phage lambda	
Gp26c	c (21363..21614)	83	9231	Gp28c	22402:22653	83	9231	100	Gp28c	22402:22653	83	9231	100	Gp28c	22402:22653	2565891..2566142	CTI08_12550	83	9231	100	Excisionase	6AMA_H		0.24 (93.44)	Streptomyces venezuelae-Mycobacterium phage Pukovnik	
Gp27c	c (21667..22353)	228	26377	Gp29c	22706:23392	228	26377	100	Gp29c	22706:23392	228	26377	100	Gp29c	22706:23392	2565188..2565894	CTI08_12545	228	26377	100	Arginine repressor	1AOY_A		0.29 (93.26)	Escherichia coli	
Gp28c	c (22595..23182)	195	21192	Gp30c	23392:24429	345	35426		Gp30c	23392:24267	291	30056		Gp30c	23392:24294	2564250..2565152	CTI08_12540	300	31348		ADP-ribosyltransferase	EKL44019.1	2e-108 (83)		Acinetobacter baumannii OIFC074 protein ACIN5074_2179	Internal repeat region causes frame shift mutation in fEg-Aba01 and size differences in others
Gp29c	c (23258..23494)	78	9110	Gp31c	24505:24741	78	9110	100	Gp31c	24343:24579	78	9110	100	Gp31c	24370:24606	2563938..2564174	CTI08_12535	78	9110	100	Antitoxin	WP_000129433.1	3e-52(100)		Acinetobacter	
Gp30c	c (23478..24113)	211	23140	Gp32c	24725:25360	211	23140	100	Gp32c	24563:25198	211	23140	100	Gp32c	24590:25225	2563436..2563954	CTI08_12530	211	23140	100	Xre-like regulator	3IVP_D		9.1e-10 (98.69)	Clostridium difficile	
Gp31c	c (24116..24406)	96	11273	Gp33c	25363:25653	96	11273	100	Gp33c	25201:25491	96	11273	100	Gp33c	25228:25518	2563026..2563316	CTI08_12525	96	11333	98.9	Hypothetical protein	WP_088741811.1	9e-65 (100)		Acinetobacter baumannii	16 aa longer N-term
Gp32c	c (24657..25349)	230	25198	Gp34c	25904:26596	230	25198	100	Gp34c	25742:26434	230	25198	100	Gp34c	25769:26461	2562083..2562775	CTI08_12520	230	25198	100	CI-like repressor	3BDN_A		3.9e-26 (99.91)	Enterobacteria phage lambda	19 aa longer N-term
Gp33	25478..25663	61	6912	Gp35	26725:26910	61	6912	100	Gp35	26563:26748	61	6912	100	Gp35	26590:26775	c (2561769..2561954)	CTI08_12515	61	6912	100	Cro repressor	3BD1_C		2.6e-8 (98.24)	Xylella fastidiosa	
Gp34	25722..26018	98	11463	Gp36	26969:27265	98	11463	100	Gp36	26807:27103	98	11463	100	Gp36	26834:27130	c (2561414..2561710)	CTI08_12510	98	11463	100	Hypothetical protein	WP_060454564.1	2e-65 (100)		Acinetobacter	
Gp35	26018..26902	294	33946	Gp37	27265:28149	294	33946	100	Gp37	27103:27987	294	33946	100	Gp37	27130:28014	c (2560530..2561414)	CTI08_12505	294	33946	100	Putative repressor	5J2Y_B		1.8e-7(98.19)	Pseudomonas aeruginosa	
Gp36	26902..28233	443	49693	Gp38	28149:29480	443	49693	100	Gp38	27987:29318	443	49693	100	Gp38	28014:29345	c (2559199..2560530)	CTI08_12500	443	49693	100	DNA helicase	2Q6T_B		1.5e-48 (100)	Thermus aquaticus-Bacillus phage SPP1	
Gp37	28230..28508	92	10477	Gp39	29477:29755	92	10477	100	Gp39	29315:29593	92	10477	100	Gp39	29342:29620	c (2558924..2559202)	CTI08_12495	92	10477	100	Hypothetical protein	WP_088741763.1	2e-58 (100)		Acinetobacter baumannii	
Gp38	28505..28978	157	18267	Gp40	29752:30225	157	18267	100	Gp40	29590:30063	157	18267	100	Gp40	29617:30090	c (2558454..2558765)	CTI08_12490	157	18267	100	HNH endonuclease	6O56_B		1.7e-11 (98.89)	Streptococcus pyogenes serotype M1	Misannotated for ABPk1
Gp39	28979..29536	185	21202	Gp41	30226:30783	185	21202	100	Gp41	30064:30621	185	21202	100	Gp41	30091:30648	c (2557896..2558453)	CTI08_12485	185	21202	100	DNA repair protein	3NA7_A		1.9e-25 (99.9)	Helicobacter pylori	
Gp40	29533..29703	56	6536	Gp42	30780:30950	56	6536	100	Gp42	30618:30788	56	6536	100	Gp42	30645:30815			56	6536	100	Hypothetical protein	ADX91738.1	1e-33 (100)		Acinetobacter baumannii TCDC-AB0715	Not annotated in AbPK1
Gp41	29703..30200	165	18965	Gp43	30950:31447	165	18965	100	Gp43	30788:31285	165	18965	100	Gp43	30815:31312	c (2557232..2557729)	CTI08_12480	165	18965	100	Antitermination protein	6JNY_B		7.6e-15 (99.41)	Enterobacteria phage Sfl	
Gp42	30281..30475	64	7433	Gp44	31528:31722	64	7433	100	Gp44	31366:31560	64	7433	100	Gp44	31393:31587			64	7433	100	Hypothetical protein	ACJ41827.2	1e-15 (97)		Acinetobacter nosocomialis	Not annotated in AbPK1
Gp43	30580..30855	91	10434	Gp45	31827:32102	91	10434	100	Gp45	31665:31940	91	10434	100	Gp45	31692:31967	c (2556577..2556852)	CTI08_12475	91	10434	100	Hypothetical protein	WP_000780217.1	4E-60 (100)		Acinetobacter calcoaceticus	
Gp44	31089..31259	56	6615	Gp46	32336:32506	56	6615	100	Gp46	32174:32344	56	6615	100	Gp46	32201:32371			56	6615	100	Hypothetical protein	AWD93141.1	6e-24 (78)		Acinetobacter phage AM106	Not annotated in AbPK1
Gp45	31252..31527	91	10313	Gp47	32499:32774	91	10313	100	Gp47	32337:32612	91	10313	100	Gp47	32364:32639			91	10313	100	HNH endonuclease	6O56_B		0.0044 (96.22)	Streptococcus pyogenes serotype M1	Not annotated in AbPK1
Gp46	31565..31783	72	8238	Gp48	32812:33030	72	8238	100	Gp48	32650:32868	72	8238	100	Gp48	32677:32895	c (2555649..2555867)	CTI08_12465	72	8238	100	Hypothetical protein	WP_000238616.1	2e-45 (100)		Acinetobacter calcoaceticus/baumannii complex	8aa in N-term
Gp47	31859..32257	132	14657	Gp49	33106:33504	132	14657	100	Gp49	32944:33342	132	14657	100	Gp49	32947:33369	c (2555175..2555573)	CTI08_12460	140	15707	100	Hypothetical protein	AGR48741.1	4e-19 (40)		Salmonella phage SPC32H	3 aa in N-term
Gp48	32273..32686	137	16019	Gp50	33520:34050	176	20723	100	Gp50	33358:33888	176	20723	100	Gp50	33385:33915	c (2554629..2555159)	CTI08_12455	176	20723	100	Similarity to NinB associated in recombination	1PC6_B		2.9e-19 (99.7)	Enterobacteria phage lambda	Intragenic deletion in g48 of fEg-Aba01 causing frame shift mutation and truncation of the 3'-end of the gene
Gp49	32726..32908	60	6585	Gp51	34040:34222	60	6585	100	Gp51	33878:34060	60	6585	100	Gp51	33905:34087	c (2554457..2554639)	CTI08_12450	60	6585	100	Hypothetical protein	SSQ43933.1	5e-38 (100)		Acinetobacter baumannii	
Gp50	32914..33207	97	11297	Gp52	34228:34521	97	11297	100	Gp52	34066:34359	97	11297	100	Gp52	34093:34386	c (2554158..2554451)	CTI08_12445	97	11297	100	Hypothetical protein	AFI96268.1	9e-66 (99)		Acinetobacter baumannii MDR-TJ	
Gp51	33137..33436	99	11354	Gp53	34451:34750	99	11354	100	Gp53	34289:34588	99	11354	100	Gp53	34316..34615	c (2553929..2554228)	CTI08_12440	99	11354	100	HNH endonuclease	6O56_B		5.3e-10 (98.61)	Streptococcus pyogenes serotype M1	
Gp52	33481..33705	74	8374	Gp54	34795:35019	74	8374	100	Gp54	34633:34857	74	8374	100	Gp54	34660:34884	c (2553660..2553884)	CTI08_12435	74	8374	100	Hypothetical protein	WP_000202128.1	2e-48 (100)		Acinetobacter baumannii	

Table S4. Detection of prophage specific sequences by PCR in the clinical strains used for phage enrichment (Table S1) using prophage-specific primer pairs (Table S2).

DNA template	Size of PCR product	
	**Aba03-F1 Aba03-R1**	**Aba03-F2 Aba03-R2**
fEg-Aba01	700	404
5542	-	-
5570	-	-
5568	-	-
5596	-	-
5597	-	-
5706	-	-
5707	700	404
5729	-	-
5933	-	-
6594	-	-
5730	-	-
5731	-	404
5907	700	404
5911	-	-
5919	-	-
5920	700	404
5923	-	-
5924	-	-
5910	-	-
6597	-	-

Table S5. Detection of prophage sequences in lysogens by PCR

**DNA SOURCE**	Primer pair				
	Aba03-F1 Aba03-R1	Aba03-F2 Aba03- R2	AbPK1-F AbPK1-R[a]	AbPK1-F Aba03-R3	Aba03-F3 AbPK1-R
fEg-Aba01	700	404	-	-	-
A. baumannii #6597	-	-	905	-	-
Lysogen 1	700	404	(905)	878	1123
Lysogen 2	700	404	(905)	878	1123
Lysogen 3	700	404	(905)	878	1123
Lysogen 4	700	404	(905)	878	1123
Lysogen 5	700	404	(905)	878	1123
Lysogen 6	700	404	(905)	878	1123
Lysogen 7	700	404	(905)	878	1123
Lysogen 8	700	404	(905)	878	1123
Lysogen 9	700	404	(905)	878	1123
Lysogen 10	700	404	(905)	878	1123

[a] The primer pair also amplified the 905 bp product from the lysogens due to the duplicated *att* site.
